# Engineered fano resonances in a compact Si_3_N_4_ photonic crystal nanobeam-microring platform for multi-cladding environments

**DOI:** 10.1038/s41598-026-35490-w

**Published:** 2026-02-05

**Authors:** Jesus Hernan Mendoza-Castro, Artem S. Vorobev, Simone Iadanza, Bernhard Lendl, Giovanni Magno, Liam O’Faolain, Marco Grande

**Affiliations:** 1 Department of Electrical and Information Engineering, Politecnico di Bari, Via E. Orabona, 4, 70126 Bari, Italy; 2 Institute of Chemical Technologies and Analytics, TU Wien, Getreidemarkt 9/164, 1060 Vienna, Austria; 3 Centre for Advanced Photonics and Process Analysis, Munster Technological University, Bishopstown, Cork, T12 T66T Ireland; 4 Tyndall National Institute, Cork, T12 PX46 Ireland; 5 Laboratory of Nano and Quantum Technologies, Paul Scherrer Institut, 5323 Villigen, Switzerland; 6 Laboratory of Integrated Nanoscale Photonics and Optoelectronics, École Polytechnique Fédérale de Lausanne, 1015 Lausanne, Switzerland

**Keywords:** Engineering, Materials science, Optics and photonics, Physics

## Abstract

The steep slope of the asymmetric Fano resonance offers potential for enhancing signal readout in compact photonic sensors across gas and liquid environments. However, achieving and controlling Fano resonance shapes on ultra-compact, fabrication-constrained platforms, particularly across variable claddings, remains challenging. We demonstrate a CMOS-compatible Si_3_N_4_ photonic platform based on a photonic crystal nanobeam (PhCN) side-coupled to a racetrack microring resonator (MRR), enabling engineered Fano resonances through passive geometric control. By varying the PhCN length and coupling gap, we systematically modulate the interference conditions that define resonance asymmetry and slope. Numerical and experimental results under both air and aqueous claddings show that the cladding-dependent modal transition, from leaky (air) to guided (liquid) backgrounds, enables robust, geometry-driven Fano behavior. A temporal coupled-mode theory model supports the results. The fabricated devices show steep asymmetric lineshapes, with ${Q}_{t}$>5$\cdot$10^3^, ER > 14dB (up to 20 dB maximum across all devices), $q$ > 0.4, and slope responsivity >5 nm^–1^(or 40–50 dB/nm), all within a compact footprint of ~40 × 34 µm^2^. The performance is comparable to similar MRR-based Fano implementations. This work provides a reproducible strategy for slope-optimized, passive Fano devices suitable for intensity-based refractive index sensing in lab-on-chip systems operating under variable cladding conditions without requiring ultra-high $Q$ or extreme ER. Thus, it serves as a design framework for future implementations.

**Supplementary Information:**

The online version contains supplementary material available at 10.1038/s41598-026-35490-w.

## Introduction

Originating from the interference between discrete resonant modes and a continuous background spectrum, Fano resonances are characterized by their distinct asymmetric lineshape^1^. These resonances have been extensively explored in photonic systems, enabling a variety of applications including all-optical switching^2^, lasing^3^, non-reciprocal pulse routing^4^, and highly sensitive detection of (bio)chemical and biological analytes^5–8^. Various Fano configurations have been implemented on Silicon (Si) and Silicon-Nitride (Si_3_N_4_) platforms, primarily via coupling between resonators with tunable coupling strengths. Among the architectures explored, photonic crystal cavities and Micro Ring Resonators (MRRs) have attracted significant interest due to their high-quality factors ($Q$), strong extinction ratios ($\mathrm{ER}$), and reduced footprints. These features support the discrete mode formation required for Fano resonance generation. Asymmetric spectral responses have been demonstrated in coupled photonic crystal nanobeam cavities and related bus waveguides^9–16^. Additional implementations include cascaded or nested MRRs^17–20^, and MRRs combined with Mach-Zehnder interferometers^21–24^. However, these configurations often face inherent trade-offs among $Q$-factors, fabrication complexity, scalability, and reproducibility. For instance, intricate device architecture demands tight fabrication tolerances, raising cost and device complexity. Additionally, larger device footprints can limit high-density integration, reducing suitability for portable lab-on-a-chip applications.

To address these challenges, assisted MRRs configurations incorporating subwavelength Fabry-Perot cavities have been proposed to control quasi-continuum modes by perturbing the effective phase and amplitude balance between resonant and non-resonant pathways^4,6,7,25–39^. These designs offer increased spectral flexibility but often remain constrained by narrow operational bandwidths, complex fabrication workflows, and limited ability to systematically navigate trade-offs between sensitivity, footprint, and spectral slope using passive design parameters alone. *Supplementary Material, Section A*, provides a detailed summary of state-of-the-art assisted-MRR approaches. This work explores the Fano resonance effect in a photonic crystal nanobeam (PhCN)-coupled MRR system, addressing the fundamental requirements for the generation of Fano shapes on gas and aqueous cladding environments. The asymmetry arises from interference between the MRR’s high-$Q$ resonant modes and bus-waveguide continuum modes, whose nature depends on the surrounding cladding: in air, the waveguide mode partially leaks through the PhCN, whereas under aqueous cladding it becomes guided due to reduced refractive index contrast, resulting in a broadband background transmission channel that effectively acts as a quasi-continuum in the Fano interference process. This interference generates sharp, asymmetric spectral lineshapes, that enable operation across both gas and liquid environments using the same platform, with leaky-wave interference in air and guided-wave interference under aqueous cladding. Compared to earlier Fano-MRR configurations, this approach offers a streamlined and fabrication-accessible geometry that enables consistent control over resonance shape and slope across cladding environments, using only passive design adjustments. The comparative summary presented in Table S1 highlights this multi-cladding compatibility.

Unlike our previous implementation based on circular perturbations in single-environment operation, the present work introduces a slot-based PhCN-MRR architecture that enables explicit control of broadband background transmission through purely geometric parameters. This additional degree of freedom allows systematic modulation of Fano asymmetry ($q$), $\mathrm{ER}$, and spectral slope across different coupling regimes and cladding environments. Rather than reporting optimized device performance, this study establishes experimentally validated design rules linking geometry, coupling strength, and cladding to the resulting Fano response. The manuscript details the design process, modeling, and simulation, followed by device fabrication and experimental validation in both air and aqueous environments. The focus is placed on how spectral slope and related parameters (e.g., $Q$, $q$, $\mathrm{ER}$) evolve with the PhCN length (${N}_{H}$) and lateral coupling gap (${g}_{y}$). While the devices studied were not optimized for maximum asymmetry, they exhibit reproducible Fano-like behavior across the full measurement range (Telecom C-band). This allows for robust experimental investigation of how moderate asymmetry influences key performance metrics.

We provide new insight into how structural tuning, particularly through slope and asymmetry control, adds a powerful degree of freedom for tailoring resonance behavior in compact photonic sensors. Within this moderate-asymmetry regime, our approach demonstrates meaningful performance improvements and application-oriented trade-offs. Our analysis revisits the generation of Fano resonances in ultra-compact Si_3_N_4_ devices, with a focus on understanding how key structural parameters, such as coupling gap, photonic crystal length, and cladding environment, influence the resonance shape and slope. Beyond experimental validation, the aim of this study is to consolidate these results into a systematic design space for PhCN-MRRs, providing a practical framework that enables researchers and designers to predictably generate Fano responses with targeted performance metrics. This approach is particularly relevant for refractive index sensors in scalable, low-loss platforms, where $Q$-factor may be constrained by fabrication tolerances or scattering-related losses rather than intrinsic material absorption. A complete list of symbols and acronyms is provided in Table 1.

**Table 1:** The list of symbols and notations.

Symbol	Description	Symbol	Description
$F\left(\omega\right)$	Fano fitting model	PTE	Partially transmitting element
${\omega}_{0}$	Resonant frequency	*N*_*H*_	Number PTE
$\varGamma$	Linewidth	*g*_*y*_	Coupling gap between MRR & PhCN
$q$	Fano asymmetry parameter	${t}_{{\mathrm{SiO}}_{2}}$ & ${t}_{{\mathrm{Si}}_{3}{\mathrm{N}}_{4}}$	Silicon oxide and Silicon nitride thicknesses
$\varDelta{\omega}_{F}$	Fano resonance signal rejection bandwidth	${n}_{{\mathrm{SiO}}_{2}}$& ${n}_{{\mathrm{Si}}_{3}{\mathrm{N}}_{4}}$	Silicon oxide and Silicon nitride refractive indexes
$\varDelta{\omega}_{L}$	Lorentzian resonance signal rejection bandwidth	${\kappa}_{x}$	Wavevector
MRR	Micro Ring Resonator	$\lambda$	Wavelength
$\omega$	Incident light frequency	$T\left(\omega\right)$	Theoretical Fano model
*R*_*MRR*_	Radius of the Micro Ring Resonator	${\gamma}_{1}$	Input waveguide decay rate
*W*_*R*_	MRR waveguide strip width	${\gamma}_{2}$	Output waveguide decay rate
*L*_*c*_	Coupling length	${\gamma}_{i}$	Intrinsic loss rate
PBG	Photonic band gap	${Q}_{t}$	Total (Loaded) $Q$-factor
PhCN	Photonic Crystal Nanobeam	${Q}_{C}$	Coupling $Q$-factor
*a*	Lattice periodicity	${Q}_{i}$	Intrinsic $Q$-factor
*W*_*s*_	Rectangular slot width	${Q}_{r}$	Free-space radiation loss
*W*_*y*_	Waveguide strip width	${Q}_{s}$	Scattering loss
SR	Slope of the resonance	${t}_{B}$	Transmission coefficient PTE
ER	Extinction Ratio	RI	Refractive Index
FOM	Figure of Merit	FSR	Free Spectral Range
DUT	Device Under Test	IPA	Isopropyl alcohol (Isopropanol)

## Principle of the proposed Fanoresonator

**Fig. 1:**
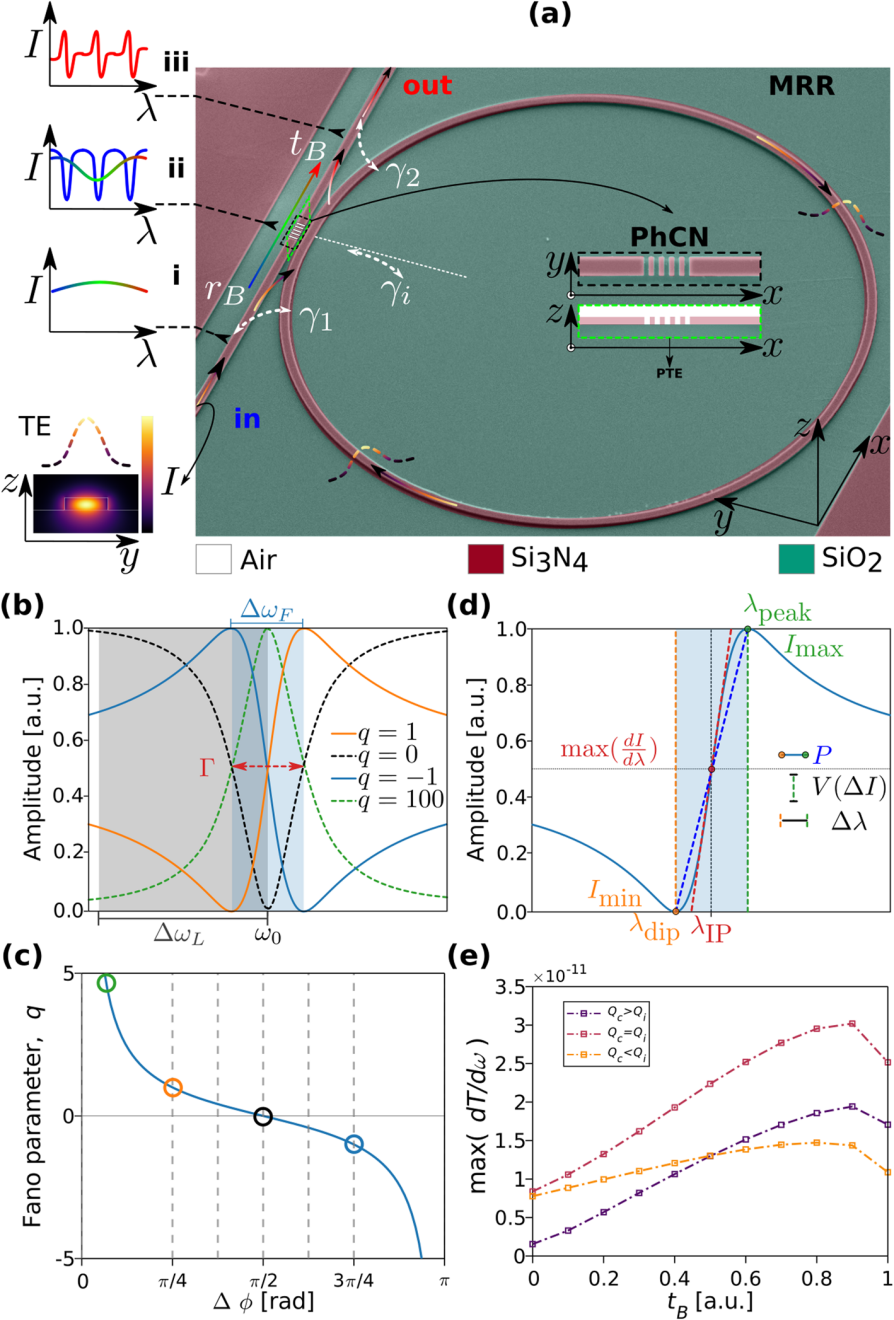
(a) Sketch of the proposed Fano cavity comprising a rectangular air-based slot Photonic Crystal Nanobeam side-coupled with a Micro Ring Resonator. The dashed gradient curve depicts the spatial mode profile (lower left) of the TE-like (${E}_{y}$) propagating mode. The rainbow, red, and blue curves represent the spectral line shapes of the propagating modes. (b) Amplitude normalized transmittance of resonances centered at ${\omega}_{0}$, with linewidth $\varGamma$ exhibiting Lorentz-like (dashed) and Fano (solid) line shapes. Signal suppression levels are indicated as $\varDelta{\omega}_{F}$ (light blue) and $\varDelta{\omega}_{L}$ (light black). The Fano asymmetry parameter $q$($-1<q<100$) spans Lorentzian, Fano, and electromagnetically induced transparency-like resonances. (c) Mapping of $q$ to the phase difference ${\Delta}\varphi$. Scatter points correspond to Fano shapes shown in (b). (d) Performance metrics of an asymmetric line shape: Slope evaluation parameter ($P$), Visibility ($V$), wavelength difference (${\Delta}\lambda$), and maximum slope at the inflection point, $\mathrm{max}\left(\frac{\mathrm{d}\mathrm{I}}{d\lambda}\right)$. (e) Extracted slope values for the distinct regimes of ${Q}_{c}$ and ${t}_{B}$, as derived from the model in Eq. (1).

The proposed Fano resonator exploits an in-plane geometry composed of a PhCN side-coupled to a MRR. The device operates based on the interference between two pathways, as shown on the sketch in Fig. 1a: (*i*) light directly transmitted through the PhCN (“continuum” pathway) and (*ii*) light circulating inside the MRR and interacting with the PhCN (“discrete” pathway). Fano resonances occur when light from both pathways interferes at the output plane (See Fig. 1a *i*,* ii*,* iii*). Due to the presence of the PTE generated by the rectangular air-based slots, the configuration enables light transmission and reflection in different proportions, implying that propagation occurs in both directions. While this article focuses solely on forward propagation, further work will explore the impact of reverse light, as recently discussed in^27,40^.

In our design, the broadband transmission modes near the PhCN’s band edge act as a continuum of states. These interact with the narrowband resonant modes of the MRR, which serve as discrete states. This interplay gives rise to a Fano resonance resulting from the interference between those two pathways. As a result, the physical channels that produce the resonant and transmitting modes are distinct (See Fig. 1a *i*,* iii*). The amplitude of the *m*-*th* mode in the MRR corresponds to the resonant frequency ${\omega}_{0}$. The energy in the MRR can either decay as intrinsic losses into the free space with an intrinsic loss rate ${\gamma}_{i}$, or it can decay into input and output waveguides with decay rates of ${\gamma}_{1}$ and ${\gamma}_{2}$, correspondingly. The transmission and reflection coefficients of the PhCN denoted as ${t}_{B}$ and ${r}_{B}$, can be tuned by adjusting the PhCN geometry. Figure 1a *ii* depicts a two-port system with mirror symmetry, which, according to the Temporal Coupled Mode Theory (TCMT) formalism^12,35,41–45^, determines the transmission behavior of the PhCN-MRR structure. Therefore, the analytical expression for the transmittance of the structure, which leads to the characteristic asymmetric Fano spectrum, is given below. A detailed derivation of Eq. (1), is provided in the *Supplementary Material, Section B*:

1 $$T\left(\omega\right)={\left|{-it}_{B}+\frac{2\sqrt{{\gamma}_{1}{\gamma}_{2}}{e}^{i({\theta}_{1}+{\theta}_{2})}}{i\left({\omega}_{0}-\omega\right)+{\gamma}_{1}+{\gamma}_{2}+{\gamma}_{i}}\right|}^{2}\mathrm{,}$$

where ${\omega}_{0}$ and $\omega$ are the frequencies of the cavity resonance (*m*-*th* mode at MRR) and the input signal. The intrinsic loss is denoted by ${Q}_{i}={\omega}_{0}/2{\gamma}_{i}$, and the coupling loss towards the bus waveguide is given by ${Q}_{c}={\omega}_{0}/2({\gamma}_{1}+{\gamma}_{2})$. These factors contribute to the overall cavity loss such that $1/{Q}_{t}=1/{Q}_{i}+1/{Q}_{c}$, can be used to represent the net dimensionless total decay rate also known as loaded $Q$-factor^46^. In practical terms, the cavity’s intrinsic loss includes also scattering loss due to manufacturing flaws (${Q}_{s}$) as well as radiation loss into free space (${Q}_{r}$). Taking into consideration a fixed intrinsic loss rate ${Q}_{i}$, it is possible to infer that the transmission line shape, described by Eq. (1), is mainly determined by the coefficients ${t}_{B}$, ${\omega}_{0}$ and ${Q}_{c}$ (*Supplementary Material*,* Section B*).

The asymmetric Fano-like-shapes can be modelled by the following equation^12,47,48^ and evaluated by the used fitting parameters:

2 $$F\left(\omega\right)={A}_{0}+{F}_{0}\frac{{\left[q+2\left(\omega-{\omega}_{0}\right)/{\Gamma}\right]}^{2}}{{1+\left[2\left(\omega-{\omega}_{0}\right)/{\Gamma}\right]}^{2}}\mathrm{,}$$

where $\omega$ corresponds to the incident light frequency, ${\omega}_{0}$ is the frequency of the cavity mode and ${\Gamma}$ quantifies the resonant linewidth. ${F}_{0}$ denotes a scaling factor and ${A}_{0}$ a baseline offset. The unitless Fano asymmetry parameter $q$ is given by $q=\mathrm{cot}\left({\Delta}\varphi\right)$, where ${\Delta}\varphi$ is the phase difference between the two interfering pathways. The calculated Fano resonances center at ${\omega}_{0}$ for different values of $q$ are presented in Fig. 1b–c. In this work, the ${Q}_{t}$ factor is calculated by the ratio ${\omega}_{0}/{\Gamma}$ from fitted parameters of Eq. (2). Fano resonances ideally exhibit a sharply asymmetric lineshape with steeper slopes near the inflection point (IP), compared to Lorentzian resonances, as shown in Fig. 1d. This means that even small shifts in resonance frequency (on the order of a fraction of the linewidth) can produce larger relative changes in transmitted or reflected intensity (${\Delta}{\omega}_{F}<{\Delta}{\omega}_{L}$), as shown in Fig. 1c. As a result, Fano resonances can be considered more sensitive in terms of amplitude modulation per unit frequency shift (i.e., steeper slopes), which directly benefits intensity-based sensing schemes^44^. In Fig. 1b, the dashed black line ($q=0$) represents a symmetric Lorentzian-like dip, where destructive interference between the discrete mode and the continuum occurs at a resonant frequency ${\omega}_{0}$. This corresponds to a special case of Eq. (2). The solid blue line ($q=1$) illustrates a blue-parity Fano resonance (the transmission minimum is blue-shifted to the maximum). The solid orange curve ($q=-1$) shows a red-parity Fano resonance (transmission minimum red-shifted)^49^. Lastly, the green dashed line. ($q=100$) depicts a Fano resonance with a very large asymmetry parameter, resulting in a nearly symmetric, Lorentzian-like lineshape. By tuning the Fano asymmetry parameter $q$, a wide range of spectral lineshapes can be realized, from symmetric Fano dips to Lorentzian peaks. Figure 1(d) illustrates characteristic features of a blue-parity Fano resonance profile^49^, which are commonly used in numerical analysis of resonance shapes, following approaches similar to that in^37^. The parameter $V$ describes the fringe visibility^6,37^ and is defined in terms of the peak and dip intensities as follow:

3 $$V=\frac{{\Delta}I}{{I}_{\mathrm{max}}+{I}_{\mathrm{min}}},$$

where ${\Delta}I={I}_{\mathrm{max}}-{I}_{\mathrm{min}}$. ${I}_{\mathrm{max}}$ and ${I}_{\mathrm{min}}$ are the normalized minimum and maximum transmission, respectively. The wavelength difference, ${\Delta}\lambda$, between peak and dip is defined as:

4 $${\Delta}\lambda=|{\lambda}_{\mathrm{dip}}-{\lambda}_{\mathrm{peak}}|,$$

where, ${\lambda}_{\mathrm{dip}}$ and ${\lambda}_{\mathrm{peak}}$ are the wavelengths at the dip and peak intensity, respectively. Here, the slope evaluation parameter of the Fano resonance is defined as follows^37^:

5 $$P=\frac{V}{{\Delta}{\uplambda}},$$

where the method was originally proposed to retrieve the complex RI ($n,k$) of glucose solutions at different concentrations, by tracking ${\lambda}_{\mathrm{dip}}$ and $10\mathrm{log}\left(P\right)$, respectively. In this work, $P$ is used to assess the slope ($\mathrm{SR}$) of the Fano resonance in the simulation analysis, where distinct Fano features are observed.

To quantify resonance sharpness, two extra slope-based metrics are used: the slope of the tangent line at the IP of the Fano profile, defined as $\mathrm{max}(dI/d\lambda),$ (Fig. 1(d)) and a practical approximation ${\Delta}I/{\Delta}\lambda$ defined as the ratio between intensity contrast and spectral width around the IP. These along with $P$, are employed in simulations to define the slope, which serves as a comparative figure of merit (FOM) across different resonance profiles. The evolution of $\mathrm{SR}$ under different regimes of ${t}_{B}$ and ${Q}_{c}$, is shown in Fig. 1(e), based on the model in Eq. (1). The supporting calculation is provided in *Supplementary Material*,* Section B*.

While all the three metrics are useful for simulations, only $\mathrm{max}(dI/d\lambda)$ is used for experimental quantifications. This ensures a rigorous, consistent evaluation of the resonance steepness. The approximation ${\Delta}I/{\Delta}\lambda$ may still appear in experimental plots as a visual aid. The IP region is particularly relevant for RI sensing, as it corresponds to a point of high intensity gradient with respect to wavelength^50^, enhancing sensitivity to small spectral shifts. The slope is influenced by ${Q}_{t}$, $q$, and $\mathrm{ER}$, which together define the sharpness and contrast of the resonance. A higher $\mathrm{SR}$ enhances signal contrast and potentially reduces the LoD, consistent with trends discussed in *Supplementary Material*,* Section B.*

## Micro ring resonator-photonic crystal nanobeam based on rectangular slots: design and simulations

The MRR (discrete state) and PhCN (continuum) are the two main elements of the Fano cavity. For the development of the fundamental components of the proposed device, a series of numerical calculations were carried out. Initially, the MRR and PhCN were studied as uncoupled devices, followed by an examination of their side-coupled interaction. All simulations and experiments in this work were intentionally performed for the fundamental TE-like mode, which provides greater fabrication tolerance and more reproducible Fano responses in the proposed device, enabling a systematic investigation of the geometric and coupling parameters governing asymmetry, slope rate, and extinction ratio across different cladding environments.

**Fig. 2:**
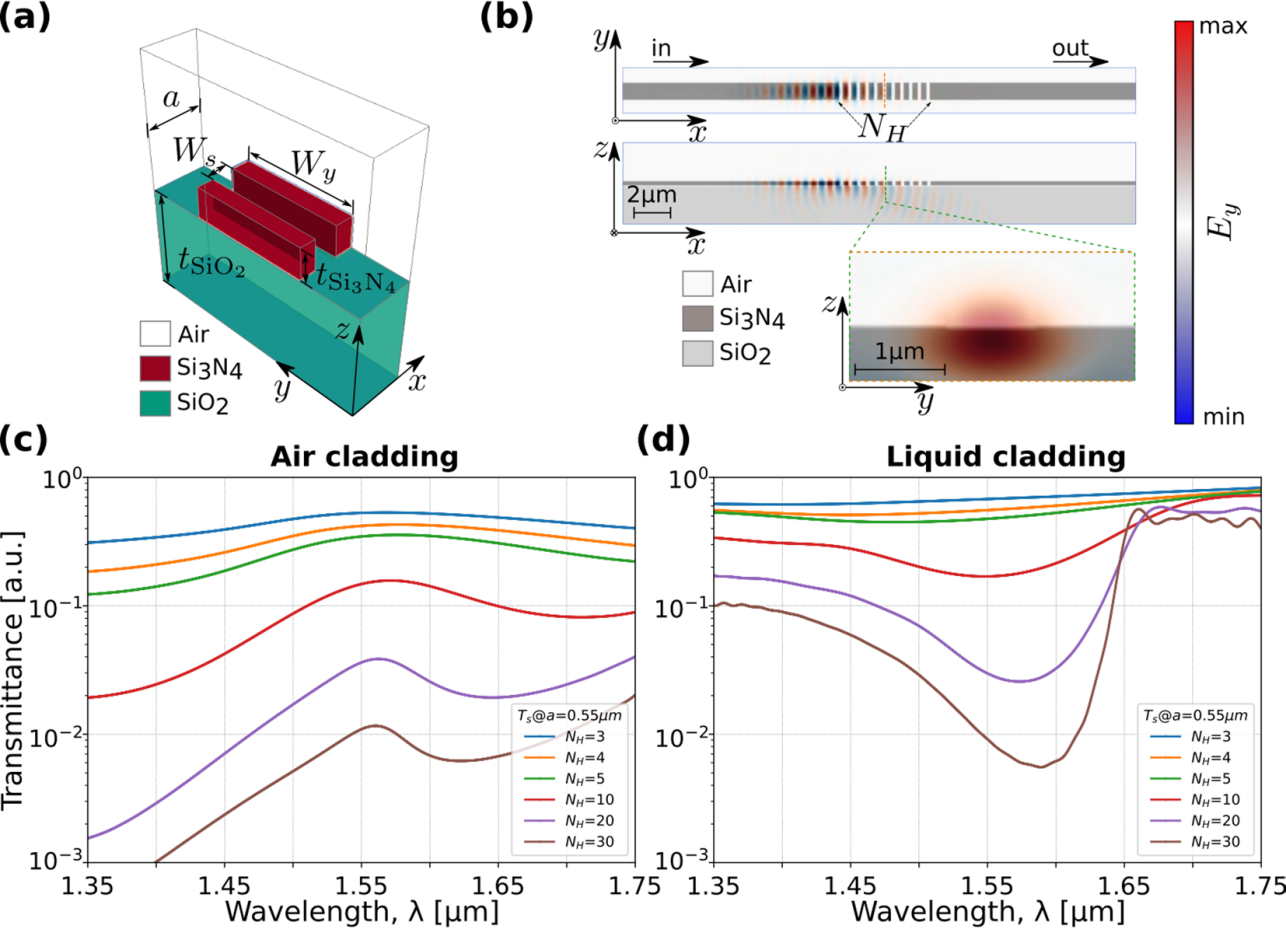
(**a**) Rectangular air-based slots PhCN unit cell. (**b**) Calculated ${E}_{y}$ distribution of a broadband pulse time evolving in the dielectric distribution of the uncoupled PhCN. (c-d) 3D-FDTD calculated transmission spectra for different numbers, ${N}_{H}$, of rectangular air-based slots with fixed $a$= 0.55 μm, ${W}_{s}$= 0.275 μm. When the upper cladding is (**c**) air and (**d**) DIW.

The transmission coefficient of the PhCN (${t}_{B}$), plays a critical role in the formation of Fano resonances. To achieve smooth control over its magnitude, the PhCN was designed as a subwavelength metamaterial structure composed of a periodic array of rectangular air-based slots. The unit cell, consisting of a Si_3_N_4_ waveguide with a rectangular slot across on top of a silica (SiO_2_) slab, is depicted in Fig. 2a–b. To gain insight into the optical response and modal properties, the dispersion relation ${\omega}_{n}\left({\kappa}_{x}\right)$ was computed assuming an infinite periodic lattice with Bloch-periodic boundary conditions along the propagation direction^51,52^. A detailed description of the computation is provided in *Supplementary Material*, *Section C*.

Although the designed structure is finite in length (see Fig. 2b), its behavior was initially analyzed based on the properties of the corresponding infinite periodic lattice, which provides insight into mode confinement and dispersion. The effective RI and mode confinement in the PhCN are strongly influenced by the RI of the upper cladding. As a result, the band edges regions of both low and high frequencies help establish a smoothly varying transmission background, facilitating tunability of ${t}_{B}$ and enabling the Fano resonance formation at the target wavelengths. The complete device response was validated using the 3D finite-difference time-domain (FDTD) simulations with perfectly matched layer boundary conditions^53^. Further details of the simulation are provided in *Supplementary Material*, *Section D*.

In Fig. 2b, the optical power of the propagating pulse experiences substantial scattering within the first three air-based slots. This scattering phenomenon is further illustrated in the side view snapshot, which captures the propagation of the transverse electric field component (${E}_{y}$) along the PhCN. The FDTD simulation reveals a partial reflection of the propagating mode, along with vertical radiation loss into the substrate and surrounding air. This behavior is consistent with the modal leakage and cutoff characteristics inferred from the dispersion analysis presented in *Supplementary Material*,* Section C.* Furthermore, the cross-sectional view in Fig. 2b provides insight into the spatial distribution of the field within the slot, located in the low-RI region, at the symmetry plane of the PhCN. The normalized transmission spectra depicted in Fig. 2c–d highlights the relationship between PhCN transmission and the number of slots ${N}_{H}$, with upper claddings of air and deionized water (DIW), respectively. An increase in the number of slots decreases the transmission level for wavelengths falling within the stop band. For the air-cladding case, the increased ${N}_{H}$ resolve the onset of a photonic band edge near 1550 nm in the transmission spectrum. While the higher-wavelength side shows a sharp spectral feature characteristic of a dielectric-band edge, the lower-wavelength side (air-band edge) continues to exhibit a gradual decline in transmittance, suggesting incomplete confinement and continued dispersion.

When assuming DIW as the top cladding material, the PBG is resolved in agreement with the behavior predicted by the dispersion curves presented in *Supplementary Material*, *Section C*. In the low-frequency band, the mode transitions from leaky to guided, leading to reduced radiation losses and an increase in transmission. For the high-frequency quasi-guided mode, although the transmission improves compared to the air-cladding case, it remains more attenuated than the low-frequency band, indicating sensitivity to cladding-induced perturbations. These numerical results also confirm the ability to tune the continuum level within the structure by adjusting the number of lattice periods ${N}_{H}$.

**Fig. 3:**
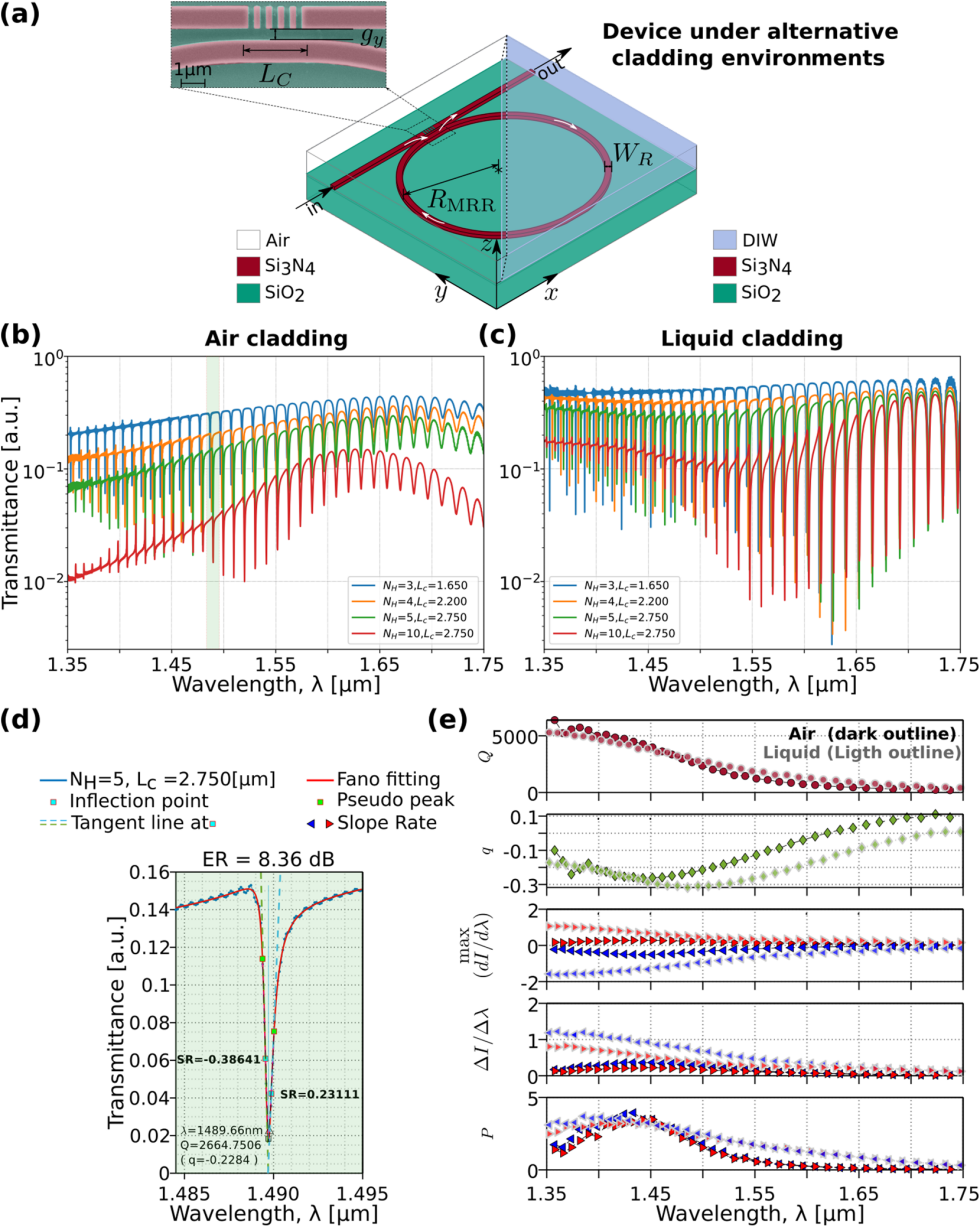
(**a**) Rectangular air-based slots PhCN-MRR designed with fixed ${R}_{MRR}$, ${N}_{H}$ = 5, and ${L}_{C}$ = ${N}_{H}a$. The upper-left inset shows the colored scanning electron microscope (SEM) image of one fabricated device. (**b**–**c**) 3D-FDTD calculated transmittance spectra of the device in (**a**) for different coupling length ${L}_{c}$ and ${N}_{H}$ with ${g}_{y}$= 0.25 μm. When the upper cladding is (**b**) air and (**c**) DIW. For ${N}_{H}$= 10, ${L}_{C}$ is fixed to 2.75 μm. (**d**) Extracted Fano resonance parameters (Eqs. 4–5) from the transmittance of the PhCN-MRR with ${L}_{c}$ = 2.75 μm and ${N}_{H}$ = 5. (**e**) Extracted parameters from the full wavelength range of the simulated spectra in (**b**) and (**c**) with ${L}_{c}$ = 2.75 μm and ${N}_{H}$ = 5. The right and left tails metrics of the resonance ($\mathrm{max}(dI/d\lambda)$, $\varDelta\, I / \varDelta \lambda$ and $P$) are drawn with a filled red and blue horizontally oriented triangle, respectively. When the upper cladding is air (dark outlined) and deionized water (light outlined).

The proposed Fano resonator based on a PhCN-MRR structure, is depicted in Fig. 3a. The racetrack MRR is characterized by its radius ${R}_{MRR}$, strip width ${W}_{R}$, and a coupling section ${L}_{C}$, tailored to match the number of lattice periods ${N}_{H}$ within the PhCN. The gap separation between the waveguides (${g}_{y}$) and the coupling length (${L}_{C}$) determines the coupling strength between the PhCN and MRR. Note that the PhCN-MRR layout has a mirror-reflection symmetry about its central axis. This symmetry is important for ensuring consistent coupling and mode interaction across the structure. The influence of ${N}_{H}$ and ${L}_{C}$ on the Fano resonance shape of the transmission has been studied using the 3D-FDTD method. Simulations considering the air-cladding and DIW-cladding cases are presented in Fig. 3b–c, respectively. The simulated spectra of the air-cladding configuration (Fig. 3b) agrees with the trend of the spectral response of the PhCN as presented in Fig. 2c. The qualitatively similar trend in Fig. 2d is also observed for the DIW-cladding case (Fig. 3c), where the spectral response evolves as ${N}_{H}$ increases. The modulation of the transmission amplitude, governed by the spectral response of the uncoupled PhCN, in turn gives rise to varying degrees of Fano asymmetry control by ${N}_{H}$. This asymmetry is further validated by the extracted resonance in Fig. 3d, which is analyzed using Eqs. (2)–(5).

For characterizing the computed spectra, $\mathrm{SR}$ on either side of the resonance is evaluated^49^. The resonance has an $\mathrm{ER}$ of 8.36 dB and a pronounced asymmetry ($q$ = $-$ 0.23), resulting in a slope ratio of ~ 1.7 between the two sides (0.3864/0.2311). Despite the asymmetry, the resonance maintains a moderate $Q$-factor (~2.7 × 10³). In Fig. 3e, we summarize the key parameters from our numerical analysis, including $Q$, $q$, $\mathrm{max}(dI/d\lambda)$, $\varDelta \,I/{\Delta}\lambda$ and $P$, for both air (dark outlined) and DIW (light outlined) cladding configurations. The analysis assumes a lossless PhCN-MRR with ${N}_{H}$ = 5, ${L}_{C}$ = 2.75 μm, and ${R}_{\mathrm{MRR}}$ = 16.63 μm. This comparison of performance metrics highlights differences in resonance characteristics between the two configurations as ${N}_{H}$ is varied. The $Q$-factor and $q$ demonstrate consistency across the two cladding types, reaching values of up to 5$\cdot$10^3^ for ${Q}_{t}$ and down to $-$ 0.3 for $q$. These trends align with the observed slope at the resonance tails, particularly near ${\lambda}_{\mathrm{dip}}$. The enhanced $Q$-factor and stronger $q$ lead to improved $\mathrm{SR}$ across all resonance modes, with a greater impact at lower wavelengths and a reduction at higher wavelengths. Additionally, $P$ exhibits notable improvement around 1550 nm, which is advantageous for the practical implementation of the proposed device design.

The parameters $\mathrm{max}(dI/d\lambda)$, $\varDelta \, I/{\Delta}\lambda$, and $P$ serve as complementary metrics for assessing the $\mathrm{SR}$, in simulation, each capturing different aspects of the resonance steepness. The SR is intrinsically linked to $q$; ideally higher asymmetry results in a steeper SR at the resonance IP. To ensure consistent quantification of the $\mathrm{SR}$ across resonances, particularly in cases approaching symmetry (i.e., when $q\to$ 0 and the lineshape becomes Lorentzian), we define a *pseudo-peak* as a reference point. It is defined by first locating the resonance minimum (${\lambda}_{\mathrm{dip}}$) and its nearest inflection point (${\lambda}_{IP}$). The spectral distance $\varDelta \lambda$ between them is then added to the IP position to create a symmetric *pseudo-peak* (see Fig. 3d). This allows us to extract paired $\mathrm{SR}$ values on both sides of the resonance in a consistent, geometry-independent way, even for nearly Lorentzian profiles.

While $P$ ($V({I}_{\mathrm{max}},{I}_{\mathrm{min}})$^37^ effectively captures slope differences in the resonance tails (e.g. Fig. 1c), it becomes less reliable for low $q$ values where peaks and dips are ill-defined. Thus, ${\Delta}I/{\Delta}\lambda$ serves as an intermediate slope estimate but it is not used in experimental analysis. In contrast, $\mathrm{max}(dI/d\lambda)$ remains the most stable and accurate figure of merit, and is thus selected for experimental validation. This approach aligns with prior methods in the literature, such as defining slope from the points where the signal reaches $0.1\cdot \mathrm{max}(dI/d\lambda)$^17^, and helps to ensure consistency and robustness in both simulation and experimental characterization.

Moreover, the dispersion characteristics and transmissivity of the quasi-guided modes, influenced by ${N}_{H}$ and the coupling between the PhCN and MRR via ${g}_{y}$, directly shape the resulting Fano profiles. This confirms that controlled Fano interference can be achieved by engineering the quasi-guided continuum, without requiring a fully resolved photonic band gap.

Additionally, complementary simulations were performed to study the effect of ${g}_{y}$ on the resonance shape for a fixed value of ${N}_{H}$ = 5 and ${L}_{c}$ = 2.75 μm. The parameter ${g}_{y}$ was varied from 0.15 μm to 0.45 μm in increments of 0.10 μm. Extra simulation details and results are provided in *Supplementary Material*,* Section D*. The observed trend aligns with model predictions at fixed ${Q}_{i}$: as ${Q}_{c}$ increases and ${t}_{B}$ remains constant, increasing ${g}_{y}$ leads to resonances with reduced visibility and extinction ratio, but steeper Fano slope (see Fig. 1(e)). Although ${g}_{y}$ is introduced as the primary tuner of the coupling strength between the PhCN and MRR, it is important to note that ${L}_{c}$ also plays a role. This opens the possibility for further analysis, where ${g}_{y}$ can serve as a coarse tuning parameter, while ${L}_{c}$ can provide fine adjustments of the coupling strength.

## Fabrication and experimental results

**Fig. 4:**
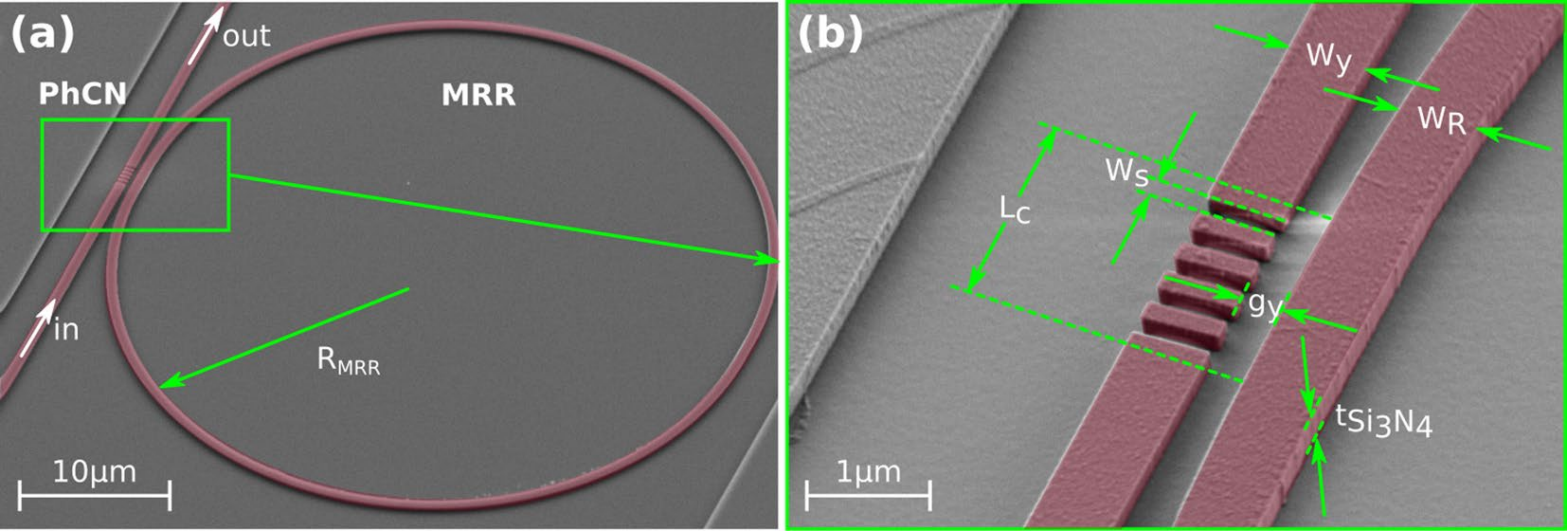
SEM images of the fabricated device on a 0.3 μm thick Si_3_N_4_ slab. (**a**) Lateral view of the fabricated rectangular air-based slot PhCN-MRR. (**b**) Detail of the coupling region with fixed ${W}_{y}$ = ${W}_{R}$ = 1.1 μm, $a$ = 0.55 μm, ${W}_{s}$ = 0.2 μm, ${L}_{c}$ = 2.75 μm and ${g}_{y}$ = 0.45 μm.

The proposed Fano resonator was implemented using conventional nanofabrication methods, with customized processing steps to attain the desired geometry. Colored scanning electron microscope images of some fabricated devices are presented in Fig. 4. The raw images are included in *Supplementary Material, Section E*.

A thermally oxidized 4” bulk Silicon wafer with a deposited layer of plasma-enhanced chemical vapor deposition (PECVD) Si_3_N_4_ (300 nm thick) was utilized. Initially, a ZEP 520 A resist layer (450 nm thick) was spin-coated onto the wafer. The desired device layouts were then defined on the resist using electron beam lithography (EBL) with a voltage of 100 kV, followed by development in a bath of n-Amyl Acetate solution for 90 s and rinsing with IPA. Subsequently, the patterns were transferred to the PECVD Si_3_N_4_ layer through an inductively coupled plasma (ICP) etching process using O_2_:CHF_3_ chemistry in an 8:42 ratio (with an etch rate of approximately ~ 72 nm/min). Resist was removed via a 5 min 100 W O_2_ plasma ashing step followed by immersion in MICROPOSIT 1165 remover for 30 min. Finally, a cleaning cycle involving Acetone, and IPA were conducted to conclude the fabrication process.

After completing the fabrication process, various devices were optically characterized using an end-fire setup, similar to the configuration described in^54^. Light from a Tunable Laser Source (SANTEC TSL-570) was coupled into the waveguide through a hybrid system consisting of fibers and spot-size converting lenses. The output from the waveguide was collected by an Optical Power meter (SANTEC MPM-212) to analyze the spectral response of the device being tested. Further details are included in *Supplementary Material*,* Section F*.

### Fano spectrum for the air-clad PhCN-MRR

**Fig. 5:**
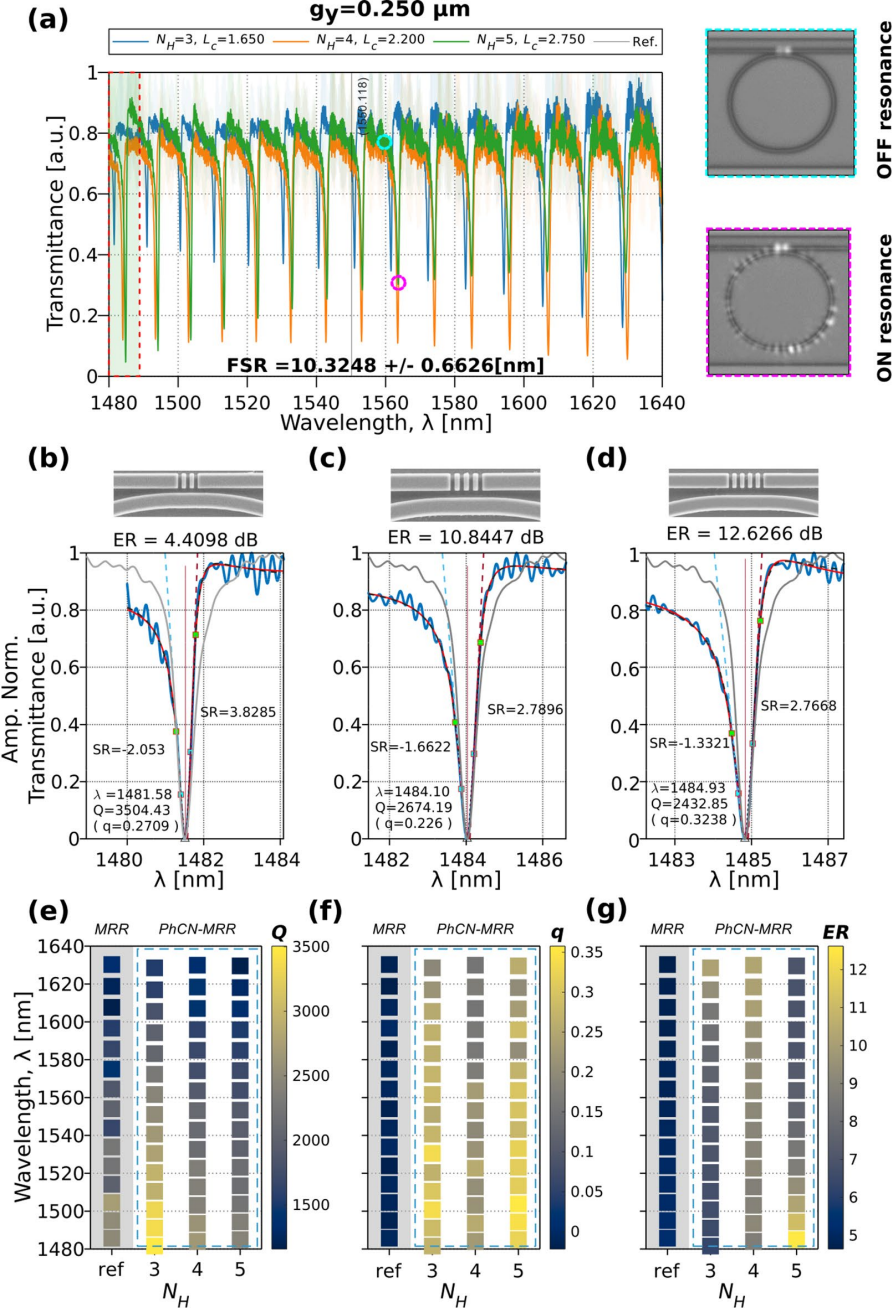
(**a**) Transmittance for the TE-like blue-parity Fano spectrum obtained for a PhCN-MRR with ${g}_{y}$ = 0.25 μm and ${N}_{H}$ = 3,4,5. Corresponding top-view scatter light images are shown for ON- and OFF-resonance states. (**b**–**d**) Experimental data (blue dots), Fano fitting (red solid line), and theoretical fitting (black dashed line) for the fabricated devices at 1483 $\pm$ 4 nm for ${N}_{H}$= 3, 4, 5. A reference resonance from a standard MRR is included (gray solid line). The SEM images from the top of the device at the PhCN-MRR coupling region. (**e**–**g**) Heat maps of the extracted metrics, ${Q}_{t}$, $q$, $\mathrm{ER}$, corresponding to the spectra in (**a**). The gray column indicates the reference values from a standard MRR while the dashed blue box highlights the values for the Fano devices under test.

The waveguide width of the feeding waveguide and MRR (${W}_{y}$ = ${W}_{R}$=1.1 μm) has been designed to support a single TE-like mode at a wavelength of 1550 nm^55^. The transmission power of the TE-like mode is optimized using polarization optics and no evidence of higher-order mode excitation was observed in either simulations or experimental transmission spectra. A series of devices were fabricated with variations in the length of the PTE, ${N}_{H}$, and the coupling gap, ${g}_{y}$, for air-based slot PhCN-MRRs, while keeping the remaining parameters constant. The characterization results validate the $Q$-factor (${Q}_{t}$) and the Fano asymmetry resonances for the air-cladding conditions. Figure 5a presents the transmission spectrum for rectangular air-based slot PhCN-MRRs with a fixed ${g}_{y}$ = 0.250 μm and varying ${N}_{H}$ = 3, 4, 5.

All transmission spectra were first normalized to the transmission of a reference straight waveguide to correct for system-level losses. This baseline normalization ensures a meaningful comparison of the $\mathrm{ER}$ between devices. Additionally, for visual clarity and to support shape-based analysis, a secondary amplitude normalization to the [0,1] range was applied to the resonance curves shown in Fig. 5b–d. While this facilitates direct visual comparison of resonance sharpness, amplitude-sensitive metrics such as $\mathrm{ER}$ are consistently extracted from the waveguide-normalized (non-rescaled) spectra to preserve amplitude fidelity. Additional discussion of slope metrics, including their dependence on amplitude normalization, is presented in a later section.

The transmittance of the devices shown in Fig. 5a exhibits periodic resonances with a FSR of 10.3248 $\pm$ 0.6626 nm. The evolution of the Fano effect for each ${N}_{H}$ configuration, as a function of wavelength, is experimentally demonstrated across the measured spectra. The strength of the Fano resonance correlates with the position of the PBG, despite its inherent dissipative behavior. The resulting modulated background (${t}_{B}\left(\lambda\right)$), as introduced in Fig. 2(c), is more pronounced at lower wavelengths where the air-band edge takes place^56^. At the same time, the shorter wavelengths may enhance coupling (${Q}_{c}$) between the PhCN and MRR. Conversely, wavelengths within the dielectric band, longer wavelengths, exhibit reduced interference interaction due to increased mode confinement. This behavior aligns with the simulation predictions shown in Fig. 2c and Fig. 3a. Figure 5a also includes far-field images of out-of-plane scattered light at the coupling region. At resonance, an annular intensity distribution is observed, consistent with the enhanced optical circulation within the MRR. Off-resonance, scattering is confined to the coupling region.

As the number of rectangular air slots in the PhCN increases, a systematic reduction of the average baseline transmission is observed across the measured wavelength range. This behavior is attributed to increased scattering and radiation loss introduced by the periodic modulation. Within the TCMT description, this effect corresponds to a reduction of the background transmission coefficient ${t}_{B}$, while the Fano interference mechanism between the microring resonance and the broadband PhCN pathway remains preserved.

Among the spectra shown in Fig. 5a, the ER for a device with ${N}_{H}$ = 4 exhibits a uniform behavior (~ 10 dB) across all measured wavelengths. In contrast, devices with ${N}_{H}$ = 3 and.

${N}_{H}$​ = 5 show increasing and decreasing trends, respectively, as the wavelength increases. Representative resonance curves near 1483 $\pm$ 4 nm are shown in (Fig. 5b–d, blue dots) with Fano model fits (Eq. (2), red lines) and the theoretical model response (Eq. (1), black dashed lines), enabling consistent comparison of ${Q}_{t}$, $q$ and $\mathrm{ER}$. The fitting range is restricted to 50% of the FSR (~ 5 nm), matching the conditions used in the simulation (Fig. 3e). Reference resonances from standard MRRs (gray lines) are included for direct comparison. The full set of extracted metrics, spanning the entire measured spectral range, is summarized in Fig. 5e–g as heat maps. Here, the gray column marks the reference MRR values, and the dashed blue box highlights the specific Fano device featured in panels (a–d).

To facilitate direct comparison of performance across different device configurations and wavelength ranges, heat maps are used to visually encode the extracted values of resonance characteristics, such as ${Q}_{t}$, $q$, $\mathrm{ER}$ from the measured spectra. In these plots, each square represents a single resonance extracted from a specific device, defined by a combination of PhCN length ${N}_{H}$ and operating wavelength $\lambda$. The vertical axis corresponds to the wavelength (typically ranging from 1480 to 1640 nm), while the horizontal axis shows the PhCN-MRR configurations, grouped by ${N}_{H}$, and labeled to distinguish between reference MRRs and slot-engineered PhCN-MRRs. The color of each square encodes the magnitude of the selected metric (e.g., ${Q}_{t}$), as indicated by the accompanying color bar. Brighter colors typically correspond to higher values (e.g., higher quality factors), while darker colors indicate lower performance. Dashed boxes group devices sharing a coupling gap ${g}_{y}$, allowing for intra and inter-group comparison of how the parameter ${N}_{H}$ and spectral position impact the resonance characteristics.

**Fig. 6:**
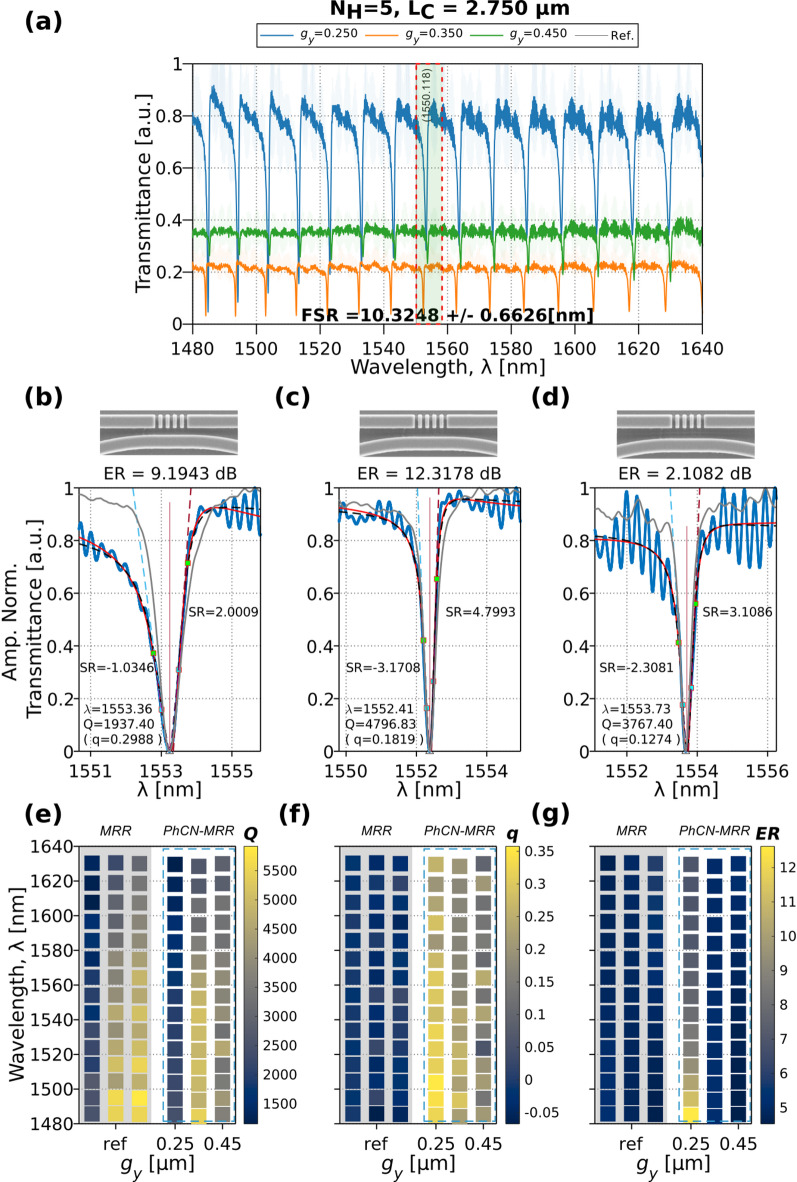
(**a**) Transmittance for the TE-like blue-parity Fano spectrum obtained for a PhCN-MRR with ${N}_{H}$= 5,${L}_{C}$= 5$a$ and varying gaps ${g}_{y}$ = 0.25, 0.35, 0.45 μm. (**b**–**d**) Experimental data (blue dots), Fano fitting (red solid line), and theoretical fitting (black dashed line) for the fabricated devices at 1553 $\pm$ 4 nm for ${g}_{y}$ = 0.25, 0.35, 0.45 μm. A reference resonance from a standard MRR is included (gray solid line). The SEM images from the top of the device at the PhCN-MRR coupling region. (**e**–**g**) Heat maps of the extracted metrics, ${Q}_{t}$, $q$, $\mathrm{ER}$, corresponding to the spectra in (**a**). The gray column indicates the reference values from a standard MRR while the dashed blue box highlights the values for the Fano devices under test.

The results in Fig. 5e–g confirm the distinct signatures of Fano resonances in air-clad PhCN-MRRs and their evolution with structural parameters (${N}_{H}$) and wavelength. Compared to the reference MRR resonance (gray trace in Fig. 5b–d), the proposed devices exhibit significantly enhanced asymmetry and extinction ratios, validating the role of Fano interference. Although the reference MRR maintains a moderate $Q$-factor (~ 3$\cdot$10^3^), its symmetric lineshape limits the slope sharpness. In contrast, the engineered asymmetry in the PhCN-MRRs results in steeper spectral transitions ($\mathrm{ER}$).

A clear inverse correlation is observed between $Q$-factor and the PTE length ${N}_{H}$: devices with shorter ${N}_{H}$ exhibit higher $Q$-factors, particularly at shorter wavelengths. In contrast, the asymmetry parameter $q$ tends to increase with ${N}_{H}$, although its behavior with wavelength is non-monotonic, $q$ decreases at ${N}_{H}$ = 4 before increasing again at ${N}_{H}$ = 5. For the $\mathrm{ER}$, different trends emerge across the three devices: at ${N}_{H}$ = 3, $\mathrm{ER}$ increases with wavelength; at ${N}_{H}$ = 4, it remains relatively stable across the spectrum; and at ${N}_{H}$ = 5, it declines as the wavelength increases. These observations highlight the interplay between structural asymmetry and spectral response, with $q$ and $\mathrm{ER}$ being the primary drivers of slope enhancement in Fano resonances.

To assess the effect of coupling strength on the resonance characteristics, we fabricated PhCN-MRRs with fixed structural parameters (${N}_{H}$ = 5, ${L}_{C}$ = 5$a$) and varying coupling gaps, ${g}_{y}$ = 0.25, 0.35, 0.45 μm. The TE-like blue-parity Fano spectra under air-cladding are shown in Fig. 6a. A top-view SEM image of the coupling region is presented in Fig. 6(b-d), revealing the PhCN-MRR interface. As ${g}_{y}$ increases, the measured resonances shift from strongly asymmetric with high extinction to narrower, more Lorentzian-like lineshapes with reduced ER, suggesting weaker modal interaction. A representative analysis of the Fano resonances near 1553 $\pm$ 4 nm is provided in Fig. 6b–d. The experimental spectra (blue dots) are fitted using Eq. (2) (red line), and theoretical fits based on Eq. (1), are overlaid (black dashed line). The extracted performance metrics across the full spectral window are summarized in Fig. 6e–g, where the left three columns (gray background) correspond to reference MRR devices for each gap, and the right three columns (dashed blue box) show the corresponding PhCN-MRR devices under test.

Figure 6e–g follows the same heat map format introduced previously, with color-coded squares representing individual resonances extracted from experimental spectra. However, in this case, the horizontal axis is grouped by coupling gap (${g}_{y}$= 0.25, 0.35–0.45 μm). The vertical axis continues to represent wavelength, and color intensity reflects the magnitude of each extracted parameter as described earlier. From the heat maps in Fig. 6e, the $Q$-factor of the standard MRRs increases with gap size, which is consistent with reduced coupling strength leading to lower radiative losses. In the PhCN-MRRs, however, the ${Q}_{t}$ peaks for ${g}_{y}$ = 0.35 μm, suggesting optimal interference between the guided mode of the MRR and the quasi-guided mode of the PhCN. In both cases, ${Q}_{t}$ tends to decrease with increasing wavelength, likely due to increased scattering at longer wavelengths.

The Fano asymmetry parameter, $q$, shown in Fig. 6f, remains near zero for the reference MRRs, indicating symmetric Lorentzian lineshapes. Small deviations from zero are attributed to fitting uncertainty and minor artifacts introduced during measurement, such as FP-like fringes or background fluctuations. In contrast, the PhCN-MRRs exhibit clear asymmetry, with $q$ values decreasing significantly as the gap increases, nearly half for the widest coupling gap, confirming the progressive suppression of interference strength in wider gaps.

The $\mathrm{ER}$ in Fig. 6g further illustrates the impact of coupling strength. As expected, $\mathrm{ER}$ generally decreases with increasing gap size due to reduced modal overlap between the PhCN and the MRR. However, a more detailed inspection reveals wavelength-dependent behavior that varies with ${g}_{y}$. For the smallest gap (${g}_{y}$ = 0.25 μm), $\mathrm{ER}$ shows a clear decreasing trend with increasing wavelength. At the intermediate gap (${g}_{y}$ = 0.35 μm), $\mathrm{ER}$ remains nearly constant across the measured spectral range, indicating a more balanced coupling condition. Interestingly, for the widest gap (${g}_{y}$ = 0.45 μm), $\mathrm{ER}$ increases slightly with wavelength, suggesting that even at weaker coupling, constructive interference can be recovered at longer wavelengths, possibly due to improved phase matching or residual modal overlap.

In summary, the air-clad measurements confirm that both the ${Q}_{t}$ and $\mathrm{ER}$ exhibit strong dependence on the structural parameters ${g}_{y}$ and ${N}_{H}$, with $\mathrm{ER}$ additionally showing wavelength-specific behavior tied to the coupling strength. The parameter $q$ consistently reflects the interference-driven nature of the resonance and remains near-zero for control MRRs, validating its reliability as a signature of Fano coupling. These findings emphasize the central role of modal interaction in shaping high-contrast, asymmetric responses under air-cladding conditions. To assess the environmental sensitivity and robustness of this behavior, we next examine the spectral response of PhCN-MRRs under DIW cladding, where the reduced refractive index contrast modifies both the effective index and scattering dynamics.

### Fano spectrum for the aqueous-clad PhCN-MRR

**Fig. 7:**
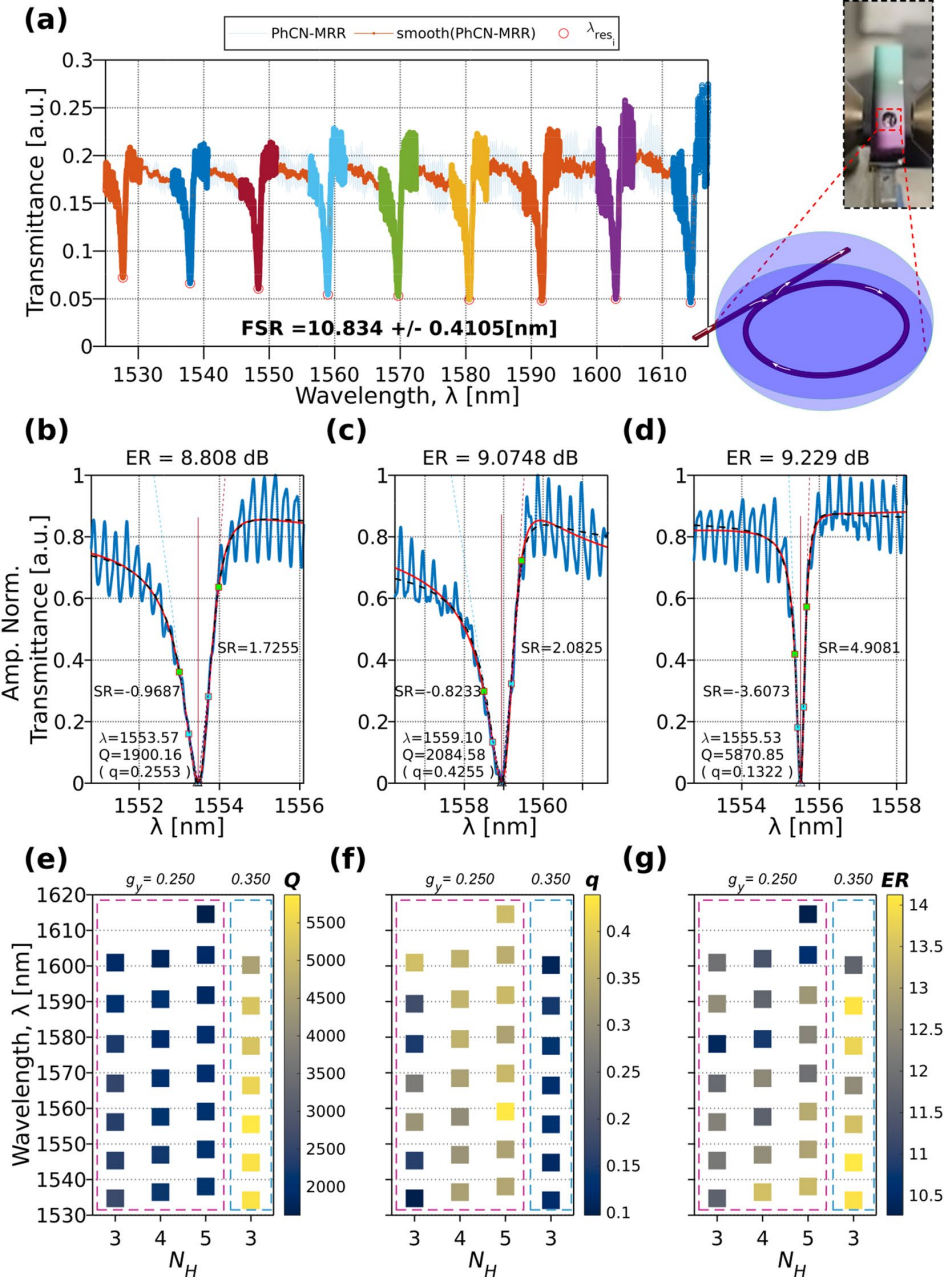
(**a**) Transmittance spectra of a slot-based PhCN-MRR clad with deionized water (*DIW*), with fixed parameters ${g}_{y}$= 0.25 μm and ${N}_{H}$=5. The inset illustrates the drop-casting process used for the DIW cladding measurement. (**b**–**d**) Experimental data (blue dots), Fano fitting (red solid line), and theoretical fitting (black dashed line) for the fabricated devices at 1554 $\pm$ 4 nm: (**b**) air-clad device with ${g}_{y}$ = 0.25 μm, ${N}_{H}$=5; (**c**) *DIW*-clad with the same geometry; and (**d**) *DIW*-clad device with ${g}_{y}$ = 0.35 μm, ${N}_{H}$=3. (**e**–**g**) Heat maps of the extracted metrics, ${Q}_{t}$, $q$, $\mathrm{ER}$, under *DIW*-cladding conditions. The red dashed box highlights the *DIW*-clad devices with ${g}_{y}$ = 0.25 μm, and the blue dashed box indicates the DIW-clad device with ${g}_{y}$ = 0.35 μm.

To evaluate the performance of the proposed PhCN-MRR under aqueous conditions, a drop-cast experiment with DIW was performed. Figure 7a shows the transmittance spectra of a PhCN-MRR with ${N}_{H}$ = 5 and ${g}_{y}$ = 0.25 μm under DIW cladding. Compared to the air-clad counterpart, a redshift in resonance and a ~ 0.5 nm reduction in the FSR is observed. Both effects are attributed to the higher refractive index of the aqueous cladding, which increases the effective and group indices of the guided mode through enhanced modal overlap with the surrounding medium^57^. Additionally, the Fano resonance exhibits enhanced asymmetry, as emphasized by the filled-color overlays on the experimental spectra (Fig. 7a, left). The drop-casting process used for the DIW cladding is schematically illustrated in Fig. 7a, right. The reduced baseline transmission observed under DIW cladding is attributed to changes in modal confinement, coupling, and radiation loss induced by the higher-index environment. Despite the increased background loss, the Fano-interference behavior is preserved and remains well described by the TCMT model.

Figure 7b–d present experimental data (blue dots), Fano fitting (red solid line), and theoretical fitting (black dashed line) of representative devices at 1554 $\pm$ 4 nm for: (b) the air-clad device with ${g}_{y}$ = 0.25 μm, ${N}_{H}$=5; (c) the corresponding DIW-clad device with the same geometry; and (d) a DIW-clad device with ${g}_{y}$ = 0.25 μm, ${N}_{H}$=3. Reference devices are not included here, as they were comprehensively covered in previous figures (Figs. 5 and 6). Notably, the dataset for the wider gap ( ${g}_{y}$ =0.35 μm) includes only ${N}_{H}$ = 3 device, limiting direct comparison across all values for this parameter.

To systematically compare resonance characteristics across a controlled set of PhCN-MRR device configurations, a series of heat maps are used. The vertical axis represents the resonance $\lambda$, while the horizontal axis groups devices by PhCN length ${N}_{H}$, with subgroupings based on the coupling gap ${g}_{y}$. For each metric ($Q$, $q$, $\mathrm{ER}$) a separate heat map is shown, enabling direct visual correlation across a shared matrix layout. The color of each square encodes the measured value of the corresponding parameter, as indicated by its color bar. This representation allows for intuitive assessment of how resonance sharpness, asymmetry, and strength evolve with device geometry and operating wavelength.

The extracted metrics heat maps in Fig. 7e–g reveal similar trends to the air-clad cases: the ${Q}_{t}$ increases with the wider coupling gap but tends to decrease with increasing ${N}_{H}$. The parameter $q$ decreases with increasing gap size but increases with ${N}_{H}$. The $\mathrm{ER}$ shows no clear monotonic dependence on ${N}_{H}$; however, some resonance regions exhibit increased $\mathrm{ER}$ with higher ${N}_{H}$, and $\mathrm{ER}$ generally improves for wider gaps. The experimental results confirm the robustness and reproducibility of the Fano resonance behavior in PhCN-MRR devices under both air and aqueous (DIW) cladding conditions. Key resonance metrics such as ${Q}_{t}$, $q$, and $\mathrm{ER}$ exhibit consistent trends across varying structural parameters (${g}_{y}$, ${N}_{H}$) and cladding environments, validating the theoretical predictions and numerical simulations.

Specifically, ${Q}_{t}$ systematically increases (> 5500) with larger coupling gaps but decreases with higher ${N}_{H}$ (longer PhCN), indicating a trade-off between modal confinement and coupling strength. The asymmetry parameter $q$ (> 0.4) shows an inverse relationship with gap size but a direct correlation with ${N}_{H}$, emphasizing the role of structural geometry in tunning the resonance lineshape. While $\mathrm{ER}$ trends are more complex, increased value (~ 14 dB) for the wider measured gap (0.35 μm) is observed, despite the reduced ${N}_{H}$ = 3. Thus, a resonance contrast responds to both structural and environmental changes. Overall, the preservation and modulation of Fano resonance characteristics in aqueous environments demonstrate the potential of PhCN-MRR devices for robust sensing applications in biologically relevant conditions.

### Slope-driven analysis for Fano lineshape engineering

Having established the consistency and tunability of resonance parameters such as ${Q}_{t}$, $q$ and $\mathrm{ER}$ across different cladding conditions and structural variations, we now shift focus to the resonance slope, $\mathrm{SR}$, at the IP. To enable systematic comparisons across devices, we introduce a slope-based figure of merit (FOM), the differential slope metric ${\Delta}\mathrm{SR}$. This metric captures the asymmetry between the spectral slopes on either side of the resonance dip. By design, ${\Delta}\mathrm{SR}\to$ 0 for Lorentzian lineshapes and increases with stronger Fano asymmetry, making it particularly valuable when full model fitting is impractical. All spectra are normalized before slope extraction, using two distinct strategies based on the figure context.

**Fig. 8:**
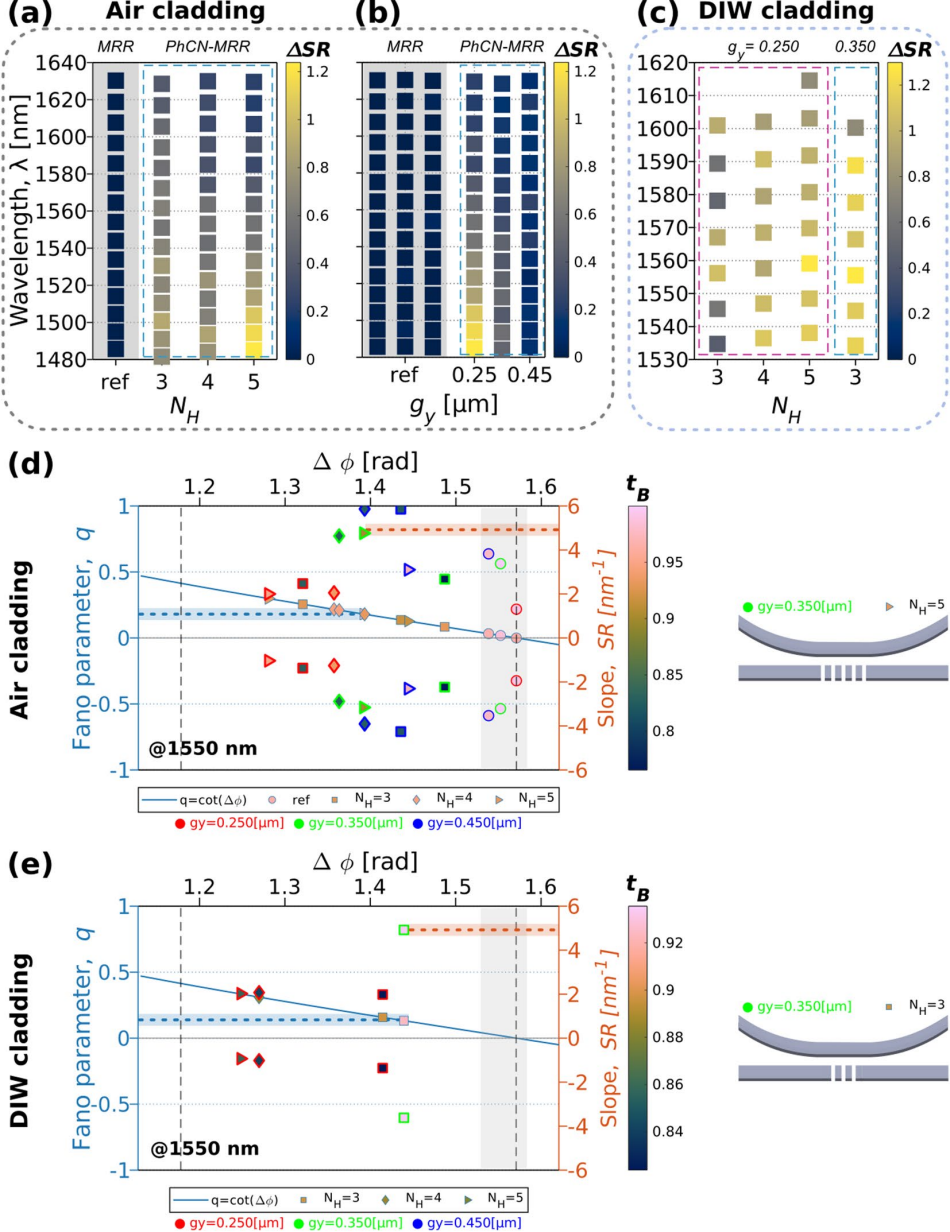
(**a**–**c**) Heat map of the slope difference ${\Delta}\mathrm{SR}$ for the PhCN-MRR devices: (**a**) air-clad devices with ${g}_{y}$ = 0.25 μm and ${N}_{H}$= 3, 4, 5; (**b**) air-clad devices with ${N}_{H}$ = 5 and ${g}_{y}$ = 0.25, 0.35, 0.45 μm; (**c**) DIW-clad devices with ${g}_{y}$ = 0.25, 0.35 μm. ${\Delta}\mathrm{SR}$ is calculated from the maximum slope values extracted from the experimental data shown in Figs. 5, 6 and 7 and uses waveguide-normalized data to reflect application-relevant response. (**d**–**e**) Fano parameter $q$ as a function of $\varDelta\varphi$ (blue solid line, left axis), with experimental data points overlaid to show the corresponding slope pairs (right axis). Marker shapes denote ${N}_{H}$, edge colors denote ${g}_{y}$, and face colors represent the fitted value of ${t}_{B}$, as indicated by the accompanying color bar. Slope values are plotted as signed pairs, with positive and negative values corresponding to the left and right sides of the resonance, respectively. Panel (**d**) shows air-clad devices; panel (**e**) shows DIW-clad devices. Gray zones correspond to Lorentz resonances $q\to$0 from reference MRRs.

In Fig. 8a–c, ${\Delta}\mathrm{SR}$ is computed from waveguide-normalized transmission spectra. This normalization reflects realistic insertion losses and baseline variations, enabling slope comparisons relevant to sensing and filtering. The plotting format follows that of Fig. 5 to Fig. 7, with vertical axes denoting $\lambda$, horizontal grouping by ${N}_{H}$ and sub-columns for each ${g}_{y}$. The color scale represents the extracted ${\Delta}\mathrm{SR}$, providing a direct visualization of slope asymmetry across device geometries and wavelengths. These panels show that: ${\Delta}\mathrm{SR}$ increases with larger ${N}_{H}$ and shorter wavelengths (Fig. 8a), indicating more pronounced Fano features. Smaller coupling gaps (e.g., ${g}_{y}$ = 0.25 μm) exhibit stronger asymmetry (Fig. 8b), while wider gaps show vanishing ${\Delta}\mathrm{SR}$, returning to Lorentz-like behavior. Under DIW cladding (Fig. 8c), ${\Delta}\mathrm{SR}$ remains non-zero even at larger gaps. The highest asymmetry arises for ${N}_{H}$ = 3, ${g}_{y}$ = 0.35 μm, highlighting how gap control takes more relevance than slot count in aqueous environments. Importantly, across all configurations, the maximum ${\Delta}\mathrm{SR}$ values saturate near 1.2, suggesting a bounded asymmetry regime for the measured devices. This behavior implies a practical upper limit for resonance imbalance, which can serve as a benchmark for evaluating and optimizing Fano resonance designs across platforms.

While ${\Delta}\mathrm{SR}$ captures asymmetry, it does not reflect the absolute slope magnitude, which is critical for sensing and modulation. To address this, in Fig. 8d–e, we fix the wavelength near 1550 nm, a relevant telecom and biosensing window, and directly correlate the left/right slopes (in [nm^− 1^]) with Fano asymmetry ($q$), phase shift (${\Delta}\varphi$), and transmission coefficient (${t}_{B}$​). The panels in Fig. 8d–e present $\mathrm{SR}$ results based on amplitude-normalized spectra, scaled to [0,1] transmission. This enables a parameter-correlated analysis that isolates the spectral shape dynamics from absolute loss and power fluctuations. Each device is represented with markers encoding ${N}_{H}$ (shape), ${g}_{y}$ (edge color), and ${t}_{B}$​ (face color). Slopes from either side of the resonance are plotted with sign, enabling direct visualization of asymmetry and magnitude.

Although the background transmission coefficient ${t}_{B}$ is treated as a real-valued parameter in the TCMT description, it represents an effective measure of the strength of the broadband interference pathway. In the physical device, modifying the PhCN geometry alters not only the amplitude of this pathway but also the phase accumulated during propagation through the perturbation region. This geometry-dependent phase difference (${\Delta}\varphi$) is not introduced as an independent variable but is implicitly captured through the evolution of the Fano asymmetry parameter $q$, which governs the observed lineshape, $\mathrm{ER}$, and $\mathrm{SR}$.

These plots offer several insights. Devices with similar slope values can emerge from different geometries, depending on the cladding. For instance, a slope of ~ 5 nm^−1^ at 1550 nm arises from ${N}_{H}$ = 5, ${g}_{y}$ = 0.35 μm under air cladding (Fig. 8 (d)). In contrast, ${N}_{H}$ = 3, ${g}_{y}$ = 0.35 μm under DIW cladding (Fig. 8e). This demonstrates a cladding-dependent design equivalence: the same optical performance (in terms of slope) can be achieved through distinct structural paths, depending on the operational environment. This flexibility is vital for designing devices under fabrication constraints.

In photonic sensing, the slope at the resonance edge is a direct indicator for sensitivity, often reported in dB/nm or RIU^−1^. Although the resonance slope is expressed in [nm^−1^], which reflects the steepness of the transmission curve in linear scale, this can be directly linked to the sensitivity in dB/nm. For example, at a normalized transmission level near 0.5 (i.e., ~ 3 dB attenuation), a slope of 5 nm^−1^ (light red in Fig. 8d–e) corresponds to a ~ 40 dB/nm change in intensity, depending on the exact ${\Delta}T$ and reference level (e.g. 1). This estimation places the PhCN-MRR devices well within the performance range of state-of-the-art Fano devices^58^. Thus, absolute slope values in our devices are not only experimentally accessible but also application relevant even with a moderate ER (e.g., measured ER in our DIW-dataset is ~ 14 dB). This contrast emphasizes that high extinction is not a prerequisite for high sensitivity: a moderate dip with steep flanks can be equally effective, especially when combined with Fano asymmetry.

Earlier high-slope responses have been demonstrated in composite interferometric architectures, such as the MRR-Mach-Zehnder system reported in^59^, achieving slope rates > 3000 dB/nm. In contrast, our side-coupled PhCN-MRR relies on local periodic perturbations to generate Fano interference within a compact footprint, with all slope measurements taken directly from fabricated devices. Although the extracted Fano asymmetry parameters are modest ($q$ < 0.4), the resulting non-Lorentzian lineshapes are sufficient to enhance slope-based sensing. This regime increases intensity slopes compared to purely Lorentzian resonances, improving sensitivity while providing robustness against fabrication and cladding variations.

The loaded $Q$ factors and asymmetry parameters reported in this work are determined by the specific PhCN geometry and coupling gaps explored here, rather than by the intrinsic material loss of Si_3_N_4_. Because the PhCN is implemented in the bus waveguide, its scattering and radiative losses are transferred to the MRR through side coupling, setting the effective loaded$Q$ of the Fano system. Different operating points within the same PhCN-MRR platform, defined by coupling strength and background transmission, can access substantially different combinations of loaded $Q$, extinction ratio, and asymmetry, without altering the underlying device concept. High- $Q$, dual-polarization Si_3_N_4_ microcavities have been reported^60^, illustrating the potential of both TE and TM operation, but the present slot-based PhCN-MRR design intentionally prioritizes TE operation for robust Fano control.

Moreover, by combining Fano asymmetry with geometric tuning, we show that device responses can be tailored to meet specific slope targets, enabling enhanced transduction even in regimes where $Q$-factor or $\mathrm{ER}$ are constrained by platform losses or tight fabrication tolerances. Figure 8a–c provides a global map of Fano asymmetry via $\varDelta \, SR$, while Fig. 8d–e offers a slope-resolved design space, linking geometry, cladding, and phase response. This dual analysis supports a cladding-aware, slope-optimized engineering strategy for photonic Fano resonators, bridging the gap between physical understanding and practical device design. While the demonstrated devices achieve meaningful asymmetry and slope enhancements, the observed $\mathrm{SR}$ is constrained by the moderate loaded $Q$ in the measured devices^24^. Maximizing $\mathrm{SR}$ in practical systems requires optimized intrinsic and coupling $Q$ factors (${Q}_{i}$ and ${Q}_{c}$) in addition to appropriate perturbation design. The implications of these trade-offs are discussed in *Supplementary Material*,* Section B*.

## Conclusions

A Fano-resonant Si_3_N_4_ photonic crystal nanobeam-microring resonator (PhCN-MRR) was designed and experimentally validated under both air-clad and DIW-clad conditions. The observed Fano resonance arises from interference between the high-$Q$ whispering-gallery mode of the MRR and broadband leaky mode supported by the slot-engineered PhCN, which effectively acts as a quasi-continuum background. This interaction is well described by the TCMT model proposed and corroborated by both spectral fitting and structural imaging.

Under air-cladding, the leaky mode partially radiates through the PhCN structure. When immersed in deionized water (DIW), the increased cladding index enhances vertical confinement and reduces radiation losses, strengthening the interference and modifying the Fano lineshape.

Comprehensive numerical and experimental analysis demonstrated how key geometrical parameters, including the coupling gap (${g}_{y}$), coupling length (${L}_{c}$), and the PhCN length (${N}_{H}$), shape the resonance slope ($\mathrm{SR}$), by tuning the total (loaded) quality factor (${Q}_{t}$), Fano asymmetry parameter $q$ and Extinction Ratio ($\mathrm{SR}$).

An optimized design achieved ${Q}_{t}$>5$\cdot$10^3^and ER > 14dB under DIW-clad conditions, exhibiting a modest Fano asymmetry parameter ($q$~0.4), with slope responsivities higher than ~ 5 nm^−1^ (e.g. corresponding to ~ 40–50 dB/nm), all within a compact footprint ( ~ 40 × 34 μm²). While $Q$ remains moderate due to bending losses associated with tight radii (e.g. 16 μm), the devices consistently show geometry-driven and cladding-robust Fano characteristics, confirming the ability of slot-based coupling interfaces to tailor resonant behavior.

We show that the Fano lineshape features, including $\mathrm{SR}$ and $q$, can be consistently tuned through ${N}_{H}$ and ${g}_{y}$, across broad spectral windows (e.g.1530–1620 nm). A slope-derived figure of merit, $\varDelta\mathrm{SR}$, was introduced to systematically capture resonance asymmetry and steepness, providing a geometry-agnostic metric to directly compare performance across devices and cladding regimes. By analyzing $\mathrm{SR}$ and $\varDelta\mathrm{SR}$ from both waveguide-normalized and amplitude-normalized datasets, we establish a unified slope-based framework for linking geometry, coupling conditions, and optical response. This framework helps identify multiple design combinations (${N}_{H},$ ${g}_{y}$, cladding) showing equivalent slopes near target wavelengths, an advantage for sensing or switching applications.

Compared to recent Si_3_N_4_ Fano demonstrations (e.g.,^33,38^), the proposed structure achieves comparable slope responsivities with added tunability across claddings, in a similar or smaller footprint (e.g.,^38^), enabled through purely geometric variation. Rather than emphasizing raw performance, this work offers a reproducible methodology to design and tune Fano responses within realistic fabrication constraints. The TCMT model further provides a minimal yet predictive design view, where background transmission ${t}_{B}$ (via${N}_{H}$) and coupling ${Q}_{c}$ (via ${g}_{y}$) emerge as key control levers, modulated dynamically by the cladding environment.

In summary, the PhCN-MRR architecture enables controlled navigation of inherent trade-offs among $\mathrm{SR}$,$ER$, $Q$, and $q$. Tuning ${t}_{B}$ through PhCN geometry simultaneously affects $\mathrm{SR}$ and $\mathrm{ER}$, allowing operating points to be selected to favor either maximal slope or improved linear range and robustness. For practical intensity-based sensing, moderate to high asymmetry combined with higher $\mathrm{ER}$ offers increased tolerance to power fluctuations and environmental noise, showing the importance of design-aware trade-off management rather than maximization of a single metric. Together, these results provide a practical framework for multi-cladding, lab-on-a-chip photonic integration.

## Supplementary Information

Below is the link to the electronic supplementary material.

Supplementary Material 1

## Data Availability

Data underlying the results presented in this paper are not publicly available at this time but may be obtained from the authors upon reasonable request.
