# Supplementary material for: Engineered fano resonances in a compact Si3N4 photonic crystal nanobeam-microring platform for multi-cladding environments
Source: Sci Rep. 2026 Feb 5;16:7347. doi: 10.1038/s41598-026-35490-w (PMC12923667; doi:10.1038/s41598-026-35490-w)
Supplement: Supplementary file 1 — Supplementary Material 1 [file 41598_2026_35490_MOESM1_ESM.pdf]

Engineered Fano Resonances in a Compact Si_3N_4 Photonic Crystal Nanobeam-Microring Platform for Multi-Cladding Environments: Supporting Information.

JESUS HERNAN MENDOZA-CASTRO [1,2,†], ARTEM S. VOROBEV [1,3,4,†], SIMONE IADANZA[3,5,6], BERNHARD LENDL[2,*], GIOVANNI MAGNO[1], LIAM O'FAOLAIN [3,4,*] AND MARCO GRANDE [1]

[1]*Department of Electrical and Information Engineering, Politecnico di Bari, Via E. Orabona, 4, 70126 Bari, Italy*
[2]*Institute of Chemical Technologies and Analytics, TU Wien, Getreidemarkt 9/164, Vienna, 1060, Austria*
[3]*Centre for Advanced Photonics and Process Analysis, Munster Technological University, T12 T66T Bishopstown, Cork, Ireland*
[4]*Tyndall National Institute, T12 PX46 Cork, Ireland*
[5]*Laboratory of Nano and Quantum Technologies, Paul Scherrer Institut, 5323 Villigen, Switzerland*
[6]*Laboratory of Integrated Nanoscale Photonics and Optoelectronics, École Polytechnique Fédérale de Lausanne, 1015 Lausanne, Switzerland*
**bernhard.lendl@tuwien.ac.at and william.whelan-curtin@mtu.ie*
† These authors contributed equally to this work

Abstract: This manuscript provides additional supporting information to complement the content presented in the main manuscript. It includes further details on both the simulated and experimentally characterized devices, offering a deeper understanding of the methodologies, results, and performed analyses.

A. Fano resonance generation in compact MRR configurations

Table S1 presents various configurations of waveguide-assisted MRRs used for generating Fano resonances. The table includes details on the device footprint, operational wavelength(s), fabrication platform, and the methods used to analyze the proposed structures. All structure configurations listed involve side-coupling of the assisting waveguide structure (e.g., Bragg grating, PhC, etc.) to a MRR.

Table S1. Representative Fano Resonator Configurations Based on Waveguide-Assisted Microring Resonators (MRRs)

Structure configuration	Footprint[a] [μm×μm]	Method[b]	λ [nm][c]	Platform[d] (thickness [μm])	Clad.[i] Cond. (s)	Sensitivity; LOD	Ref.
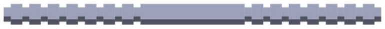	~16.4×16.4	Exp.	1.55	Si (0.220)	SiO_2	-	[1]
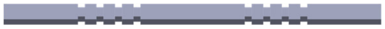	~20.5×20.5	Exp.	1550	Si (0.220)	SiO_2	-	[2]
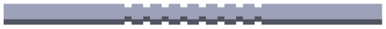	~140×121	Exp.	1550	Si (0.250)	[f]SiO_2	-	[3]
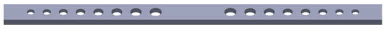	~13.2×13.2	Sim.	1550	Si (0.220)	DIW/Bio	-	[4]
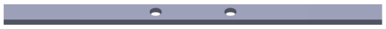	~161×161	Exp.	1550	Si (0.220)	Air	-	[5]
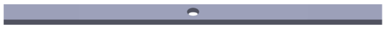	~61×61	Exp.	1550	Si (0.220)	Air	-	[6,7]
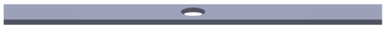	~151×151	Exp.	1550	Si (0.220)	Air	-	[8]
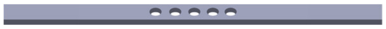	~40×34	Exp.	1310, 1550	*Si_3N_4* (0.300)	Air	-	[9]

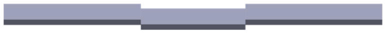	>~31×31	Exp.	1550	Polymer (-)	[g]DIW, Glucose	n/a; 0.24 mg/mL	[10]
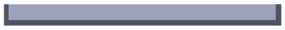	>~10×10	Exp.	1550	Si (0.230)	[f]Air	-	[11]
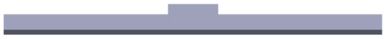	~61.2×61.2	Exp.	1550	Si (0.220)	SiO_2	-	[12]
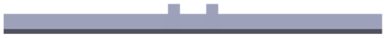	~21×21	Exp.	1550	Si (0.220)	SiO_2	-	[13]
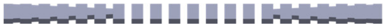	~11.5×11.5	Exp.	1550	Si (0.220)	[g]DIW, Glucose	363 nm/RIU; n/a	[14]
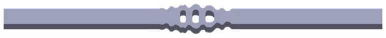	-	Exp.	1550	Si (0.220)	Air	-	[15]
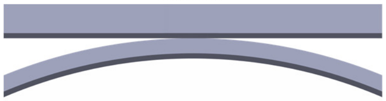	~310×304	Exp.	1550	Polymer (-)	[g]DIW Glucose	1120/RIU[h]; n/a	[16]
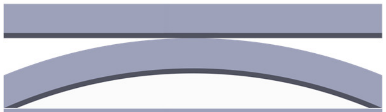	~12000 × 12000	Exp.	978	*Si_3N_4* (0.050)	SiO_2	-	[17]
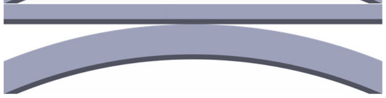	~302×302	Exp.	1550	$LiNbO_3$ (0.380)	Air	-	[18]
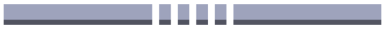	**~40×34**	**Exp.**	**1550**	***Si_3N_4* (0.300)**	**Air, DIW**	111nm/RIU	[e]

[a] Footprint, estimated area considering the radius, coupling gap and waveguide width.
[b] Study done by simulation (Sim.) and/or experiment (Exp.).
[c] Wavelength (λ) expressed in nm.
[d] Fabrication platform: Silicon (Si), Silicon Nitride (Si_3N_4), Lithium Niobate ($LiNbO_3$).
[e] This work.
[f] Assumed
[g] Sensing experiment performed (e.g. aqueous sample analyte)
[h] ΔV (visibility change) transduction
[i] Cladding Condition: Surrounding medium of the device reported
[n/a] Not available / Not explicitly reported.

Representative refractive-index sensitivity for the analyzed devices is S~110-115 nm/RIU, estimated from measured resonance shifts using glucose solutions. This is slightly lower than the theoretical bulk sensitivity (~140 nm/RIU for DIW) and consistent with our Si_3N_4 PhCN-MRR studies, illustrating the sensing potential of slot-based architecture across air and aqueous claddings. The reported sensitivities were extracted for studies compatible with liquid sensing approaches performing bulk aqueous RI measurements.

B. TCMT formalism for the derivation of the Fano model

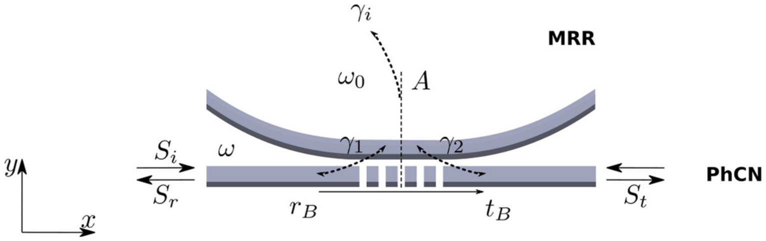

Fig. S1. Model sketch of the proposed Fano cavity comprising a slot-based PhCN side-coupled with a MRR. The vertical dashed line represents the mirror symmetry plane.

An analytical expression for the transmittance of the proposed structure is derived using the temporal coupled-mode theory (TCMT) [19,20]. For a two-port system with mirror symmetry

(see Fig. S1), and a single resonator, the incoming light with amplitude $S_i = e^{-i\omega t}$, leads to a resonant field amplitude for the *m-th* mode in the steady state given by $dA/dt = -i\omega A$. Thus, the intracavity field amplitude $A(\omega, t)$ of the PhCN-MRR could be described as follows:

$$S_t = -it_B S_i + \sqrt{2\gamma_2} e^{i\theta_2} A, \tag{S1}$$

$$\frac{dA}{dt} = -(i\omega_0 + \gamma_1 + \gamma_2 + \gamma_i)A + \sqrt{2\gamma_1} e^{i\theta_1} S_i, \tag{S2}$$

where ω is the incident frequency, ω_0 is the resonant frequency of the cavity, and γ_i, is the intrinsic loss rate of the MRR. The decay rates for the input and output coupling for PhCN-MRR are given by γ_1 and γ_2, correspondingly. S_t (S_r), represents the transmitted (reflected) wave amplitudes. The phase coupling coefficients of the coupling in/out ports of the MRR read as $e^{i\theta_1}$ and $e^{i\theta_2}$. Note that it is the interference of the two terms in equation (S1) that gives rise to the Fano resonance lineshape. Thus, the power transmission of the proposed PhCN-MRR, $|S_t/S_i|^2$ can be derived as follows:

$$T(\omega) = \left| -it_B + \frac{2\sqrt{\gamma_1\gamma_2} e^{i(\theta_1+\theta_2)}}{i(\omega_0 - \omega) + \gamma_1 + \gamma_2 + \gamma_i} \right|^2. \tag{S3}$$

Because of energy conservation, r_B and t_B should satisfy $|r_B|^2 + |t_B|^2 = 1$, $r_B^* t_B + r_B t_B^* = 0$. Furthermore, time-reversal symmetry requires that $\kappa_j = \sqrt{2\gamma_j} e^{i\theta_j}$ (j=1,2). Thus, from the values of t_B, γ_1 and γ_2 using the requirements of energy conservation and time reversal the phase terms $e^{i\theta_1}$ and $e^{i\theta_2}$ can be determined as follows:

$$e^{i(\theta_1+\theta_2)} = \frac{it_B\sqrt{\frac{\gamma_2}{\gamma_1}}}{r_B e^{-i2P\theta_1} + 1}, \tag{S4}$$

where P=$\pm$1 for the parity of the resonance [21]. Assuming θ_2=θ_1, γ_2=γ_1 equation (S3) and (S4) can be re-expressed as follows:

$$T(\omega) = \left| -it_B + \frac{2\gamma_1 e^{i2\theta_1}}{i(\omega_0 - \omega) + 2\gamma_1 + \gamma_i} \right|^2, \tag{S5}$$

$$e^{i(2\theta_1)} = \frac{it_B}{r_B e^{-i2P\theta_1} + 1}. \tag{S6}$$

For P=$\pm$1 equation (S5) can be resolved as follows:

$$T(\omega) = \left| -it_B + \frac{2it_B\gamma_1}{(r_B e^{\mp i2\theta_1} + 1)(i(\omega_0 - \omega) + 2\gamma_1 + \gamma_i)} \right|^2, \tag{S7}$$

whereby Euler equivalence results

$$T(\omega) = \left| -it_B + \frac{2t_B\gamma_1 i}{\left(\frac{r_B}{\cos(2\theta_1) \mp i\sin(2\theta_1)} + 1\right)(i(\omega_0 - \omega) + 2\gamma_1 + \gamma_i)} \right|^2. \tag{S8}$$

Considering the equivalence $\cos(2\theta_1) = -r_B$, equation (S8) simplifies to

$$T(\omega) = \left| -it_B - \frac{2t_B\gamma_1 i}{\left(\frac{r_B}{r_B \pm i\sin(2\theta_1)} + 1\right)\left(i(\omega_0 - \omega) + 2\gamma_1 + \gamma_i\right)} \right|^2. \tag{S9}$$

Assuming $\sin(2\theta_1) = \pm t_B$ for equation (S9) leads to the following expression:

$$T(\omega) = \left| -it_B + \frac{2\gamma_1 i(t_B \pm r_B i)}{i(\omega_0 - \omega) + 2\gamma_1 + \gamma_i} \right|^2. \tag{S10}$$

By applying the relation $r_B = \sqrt{1 - t_B^2}$, to equation (S10) we obtain:

$$T(\omega) = \left| -it_B + \frac{2\gamma_1 i(t_B \pm \sqrt{1 - t_B^2} i)}{i(\omega_0 - \omega) + 2\gamma_1 + \gamma_i} \right|^2. \tag{S11}$$

Accordingly, we derived:

$$T(\omega) = \left| -it_B + \frac{2\gamma_1 (it_B \mp \sqrt{1 - t_B^2})}{i(\omega_0 - \omega) + 2\gamma_1 + \gamma_i} \right|^2. \tag{S12}$$

where equation (S12) corresponds to a simplified expression of equation (1) in the main manuscript. Thus, equation (S12) reveals that the transmission characteristics of the modeled system are predominantly influenced by the parameters. t_B and γ_1 (Q_c), provided that the intrinsic loss rate, γ_i (Q_i), is usually fixed.

In a MRR, for a given round-trip loss, there exists a critical distance, $g_{y_{\text{crit}}}$, between the bus waveguide and the ring resonator (see Fig. S2(a)), at which the transmission at the resonance wavelength drops to zero, and the phase undergoes an abrupt shift of π radians, a condition known as critical coupling [22]. When the bus distance, g_y is shorter than $g_{y_{\text{crit}}}$ the MRR is said to be overcoupled, whereas if the distance is longer, it is undercoupled.

In a Fano system, the condition $Q_i = Q_c$ (rate matching) does not produce zero transmission. For the normalized Fano response of equation (S12), this condition yields a transmission minimum of 0.25. Complete extinction occurs only when rate matching is combined with appropriate asymmetry (q) and background transmission (t_B), which together determine the depth and shape of the Fano resonance. The transmission minimum therefore results from the interplay of rate matching, setting the interference condition along with the q and t_B values, shaping the lineshape and maximum extinction. This formulation aligns the TCMT model predictions with experimental observations and provides practical guidance for engineering Fano lineshapes and slope characteristics.

Based on this approach we analyzed the modeled system in the three coupling scenarios and their influence on the resonance slope. Consequently, by considering a PhCN-MRR with ω_0= 193.41 THz (1.55 μm) and Q_i, Q_c and Q_t coefficients $10\cdot10^3$, and $2\cdot10^3$, $1.67\cdot10^3$, respectively, one can analyze the influence of t_B on the optical response. Note that $1/Q_t = 1/Q_i + 1/Q_c$. Fig. S2 presents the calculated transmission based on equation (S12) and the corresponding derivatives for $t_B$values varying from 0 to 1 in steps of 0.1.

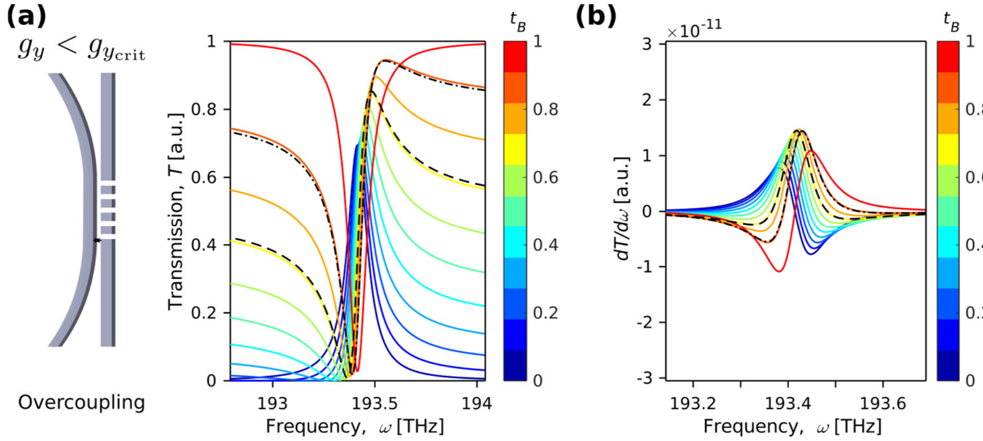

Fig. S2. (a) Power transmission of the modeled system for $t_B \in [0, 1]$ for an overcoupling condition ($Q_c < Q_i$). (b) The corresponding first derivative. The black dashed line and black dash-dotted line represent $t_B = 1/\sqrt{2}$ and $t_B = 0.9$, respectively. The horizontal axis zoomed in to highlight details near the resonance frequency.

The calculated spectra shown in Fig. S2(a) (black dashed line), highlight the spectral shape associated with a value $t_B = \frac{1}{\sqrt{2}}$, at which theoretical minimum extrema separation is expected [21]. Additionally, Fig. S2(a) includes a black dash-dotted line representing the spectrum for t_B=0.9, which exhibits the steepest slope in this example. An unambiguous Fano resonance with a steeper slope, compared to the Lorentzian profile, is observed for a decreased t_B value. Similarly, Fig. S2(b), clearly shows the transition from two extrema to one, associated with the dominance of one slope of the Fano profile over the adjacent one. The zero-crossing in the first derivative, shown in Fig. S2(b), further highlights the red shift observed in Fig. S2(a), related with the parity of the mode P (equation (S4)). Moreover, the Fano shapes that correspond to the values of t_B=1/√2 and t_B=0.9, display nearly identical slopes, as seen in Fig. S2, despite differences in resonance intensity and extinction ratio (ER). The trend in resonant intensity maintains a minimum value of approximately 0.7, while transitioning through all asymmetric shapes induced by t_B.

Consequently, a system operating under critical coupling condition ($Q_c = Q_i$) with coefficients $Q_i = Q_c = 10 \cdot 10^3$ and $Q_t = 5 \cdot 10^3$, is modeled. The computed spectra are presented in Fig. S3, along with their corresponding first derivative. Fig. S3(a) illustrates the transition from a Lorentzian dip resonance ($t_B = 1$), to a peak-like shape ($t_B = 0$). The inherently narrower dip indicates a higher slope for the Lorentz shape compared to the previous regime ($Q_c < Q_i$). Additionally, Fig. S3(b), highlights the steepest side of the Fano resonance generated when the t_B is reduced by 10% ($t_B = 0.9$). Moreover, the intensity difference at the extrema points drops significantly to 0.25 while decreasing the value of t_B.

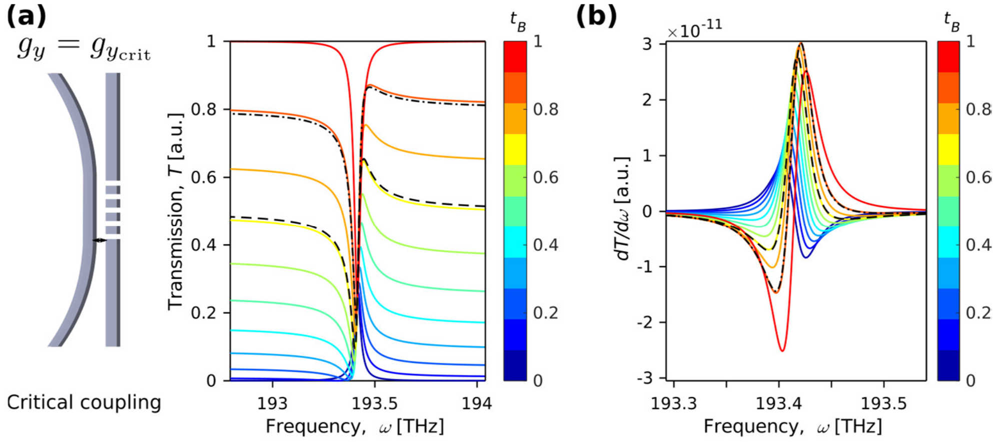

Fig. S3. (a) Power transmission of the modeled system for $t_B \in [0, 1]$ for a critical coupling condition ($Q_c = Q_i$). (b) The corresponding first derivative. The black dashed line and black dash-dotted line represent $t_B = 1/\sqrt{2}$ and $t_B = 0.9$, respectively. The horizontal axis zoomed in to highlight details near the resonance frequency.

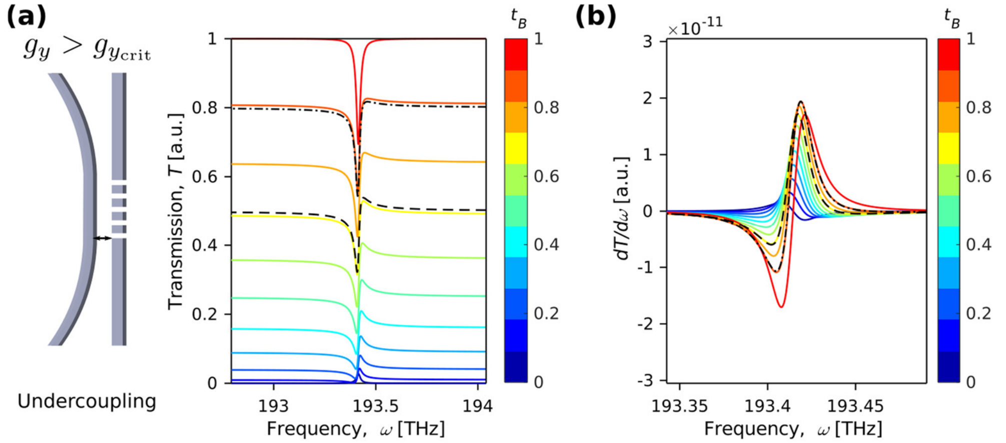

Fig. S4. (a) Power transmission of the modeled system for $t_B \in [0, 1]$ for an undercoupling condition ($Q_c > Q_i$). (b) The corresponding first derivative. The black dashed line and black dash-dotted line represent $t_B = 1/\sqrt{2}$ and $t_B = 0.9$, respectively. The horizontal axis zoomed in to highlight details near the resonance frequency.

Operation in the undercoupling condition ($Q_c > Q_i$) is analyzed for the modeled system, with $Q_i = 10 \cdot 10^3$, $Q_c = 50 \cdot 10^3$ and $Q_t = 8.33 \cdot 10^3$. The computed spectra, seen in Fig. S4(a), display the narrowest resonance for t_B=1, compared with the two previous cases. Moreover, the ER of the Fanoshape almost disappeared. Fig. S4(b) further reveals the presence of a steep slope in Fano shape, though it becomes less pronounced compared to overcoupled and critically coupled regimes. Despite the increased asymmetry introduced by the varying values for t_B, the shallower resonance intensity reduces the figure of merit of the Fanoshapes.

The resonance intensity and slope play a critical role in refractive index sensing applications. Specifically, the slope at the inflection point of a resonance determines the sensitivity of a potential refractive index transducer, while the amplitude difference between the resonance extrema points is proportional to the transducer's dynamic range. The ER can be defined as ER = $10\log_{10}(T_{\max}/T_{\min})$, and the slope at the inflection point of a Lorentzian resonance can be expressed as $\max\left(\frac{dT}{d\omega}\right)\Big|_{t_B=1}$. To ensure consistency and comparability across the computed resonance profiles, shown in Fig. S2, Fig. S3, Fig. S4, three metrics were calculated: Visibility, Slope evaluation parameter and ER. For visibility, resonance extrema points were

normalized such that the midpoint of the maximum and minimum transmission values were centered at 0.5. For ER, a vertical shift was applied to all transmission values such that a maximum transmission value of 1 is obtained. These adjustments allow for robust interpretation of resonance features, independent of baseline offsets or numerical instabilities. Furthermore, they address artifacts commonly observed during the measurement of fabricated devices such as different insertion losses for different devices.

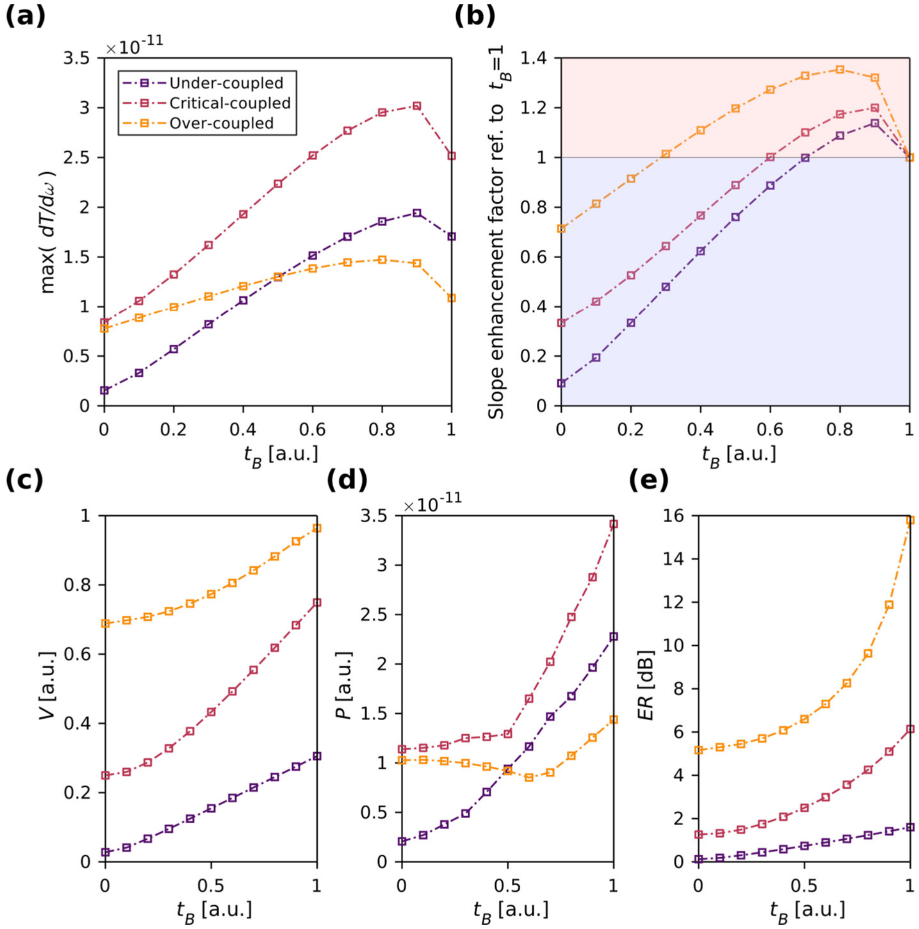

Fig. S5. (a) Maximum at the first derivative of the power transmission of the modeled system for $t_B \in [0, 1]$. (b) The slope enhancement factor is calculated as the ratio $\max\left(\frac{dT}{d\omega}\right) / \max\left(\frac{dT}{d\omega}\right)\Big|_{t_B=1}$ (c) Visibility (V), (d) Slope evaluation parameter (P) and (e) ER of each resonance profile.

Fig. S5(a) presents the extracted maximum slopes in the three analyzed regimes. The critical coupled case exhibits the highest slope for all the values of t_B. In contrast, the undercoupled regime provides a steeper slope than the overcoupled case when $t_B > 0.5$. This trend reverses for $t_B < 0.5$, where the overcoupled case surpasses the undercoupled one. Notably, for $t_B = 1$, the Lorentzian resonance demonstrates a relatively high slope compared to adjacent values of t_B (e.g $t_B < 0.7$), particularly within the range where the optimal t_B for enhanced slope is located. Therefore, Fig. S5(b) quantifies the enhancement factor of the steepness, normalized to the slope obtained in the Lorentz resonance ($t_B = 1$). A horizontal line in Fig. S5(b) indicates the baseline slope of the Lorentzian resonance, highlighting instances where Fano and Lorentz resonances exhibit identical slopes. Values above this line correspond to slope amplification, while below represents attenuation. The range of t_B for which enhancement or attenuation

occurs is determined by the intersection points with the horizontal line. Notably, this range expands as Q_c decreases. Thus, Fig. S5(b) reveals that the overcoupled regime offers the broadest range of t_B values for slope enhancement, despite exhibiting the lowest slopes across all regimes. Conversely, the critical coupling regime, while achieving the highest slope, demonstrates a reduced range of t_B values for enhancement. To further understand these behaviors, resonance intensity is analyzed by means of Visibility and ER.

Fig. S5(c) shows that the visibility is highest in the overcoupled regime. Across all regimes, the Lorentzian resonance exhibits the highest maximum extrema intensity difference. In comparison, the critical coupling regimes show a ~20% decrease in visibility for t_B = 1, while the undercoupled regime experiences a reduction approximately twice as large. Additionally, a saturation region is observed for low t_B values in both the overcoupling and critical coupling regime, followed by a common linear-like trend. The ER, shown in Fig. S5(e), reveals a direct proportional reduction of the ratio as t_B decreases, while also highlighting differences across the coupling regimes.

In the Fig. S5(d) a summary of the calculated slope evaluation parameter P is presented. The parameter P show a linear trend for t_B > 0.5, showing that resonances approaching to the critical coupling regime offer superior performance. This linearity also reflects the interdependence of P with the spectral visibility, ER. Interestingly, the optimal P values in each regime correspond to P =1, which represents the conventional Lorentzian resonance. This is particularly important given that the inherently dissipative nature of the PhCN-MRR system, could lead to reduced amplitudes, that turn into lower P. The result suggests that reduced asymmetric Fano profiles could lead to better performance.

Given that Q_c and t_B are critical parameters influencing the Fano generation, Fig. S5(c-e) provide a useful parameter space for designing a PhCN-MRR. A trade-off between the steepness of the resonance and the maximum extrema separation can be achieved in the critical coupling regime with t_B~0.9. However, achieving this condition is often experimentally challenging. The over-coupled regime, despite its shallowest slope, offers a broader range of $t_B$values for slope enhancement. Furthermore, its relatively high ER ensures a larger dynamic range, making it a more practical choice for experimental implementation as RI transducer in realistic conditions such as high-performance liquid chromatography. Conversely, while the undercoupled regime offers a reasonable slope when compared to the critical coupling regime, the narrow range of t_B values required for slope enhancement presents significant practical challenges. Achieving such enhancement through the Fano shape in this regime is therefore less feasible. It should also be noted that the analysis assumes fixed Q_i, while varying Q_C and t_B. Experimentally, the values of Q_t, Q_i and Q_c can be obtained as discussed in [22,23]. Furthermore, t_B is engineered through the design of the PhCN.

C. Band structure simulations from the PhCN varying cladding refractive index

The periodic arrangement of dielectric elements, such as rectangular slots, forms an array of Rayleigh scatterers [24]. It is well established that light propagation through a periodic slab waveguide is determined by its dispersion relation, as illustrated in Fig. S6. From this analysis, three distinct regimes can be identified: subwavelength-guided wave propagation, Bragg reflection, or the radiation regime.

Although lossless mode propagation is achievable in the deep-subwavelength region, it poses significant manufacturing challenges. Conversely, features designed to align with the transition region, between the subwavelength and Bragg regimes, offer a more practical balance of performance and manufacturability. In k-space, this transition region lies adjacent to other regimes, meaning small variations in periodicity can introduce additional spatial frequencies in the subwavelength structure. These variations may result in undesired losses due to reflection

and radiation, highlighting the importance of precise design and fabrication in this regime. Therefore, a perturbed periodicity can lead to additional losses in the realization of the desired t_B value.

The band diagram $\omega_n(\kappa)$ is calculated for propagation along x direction (κ_x) using Bloch-periodic boundary conditions [25], as illustrated in Fig. S6(a), where n denotes the band number [26]. By convention, the first band is peaked in the dielectric (1) and the next one in the air (2). The symmetry of the considered TE-like mode corresponds with the even band (fundamental mode), for which the mode profile has the fewest nodes and the lowest frequency. The air light line (black dashed) and the SiO_2 (cyan dashed) light line are added in Fig. S6(a) to assess the guided or leaking regime of the calculated bands [24,26].

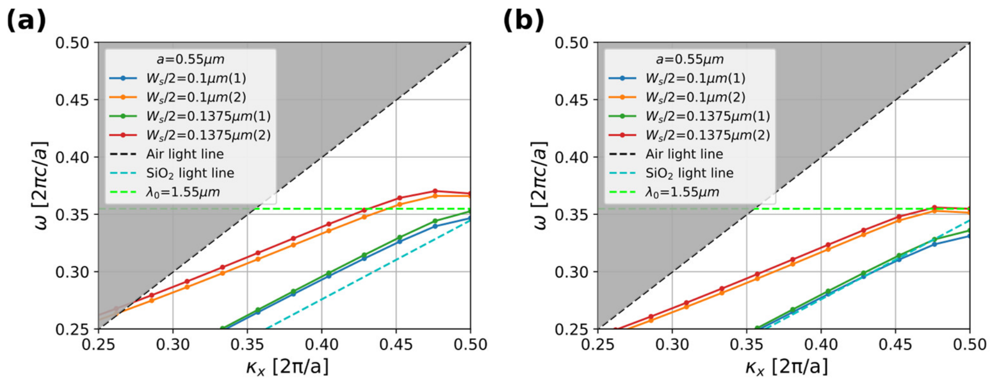

Fig. S6. (a-b) Band diagram of the TE-like guided modes, with the lowest two bands (1,2) shown for PhCN with large (W_s = 0.275 µm) and small (W_s = 0.2 µm) slot width for a fixed periodicity a = 0.55 µm. When the upper cladding is (a) air and (b) DIW. Frequencies corresponding to extended states propagating on cladding (e.g. air) above the light line shaded in gray.

Fig. S6(a) presents the calculated band diagram for a unit cell with periodicity a = 0.55 µm and slot width W_s = 2(0.1;0.1375) µm when the upper cladding is air. Increasing the slot width will shift the quasi-guided modes towards higher frequencies (lower wavelengths). Additionally, the notorious change in the width of the PBG exhibited in Fig. S6(a), indicates that in both cases, the first quasi-guided band is around the target wavelength of 1550 nm. In contrast, Fig. S6(b) shows the dispersion relation of the unit cell when the upper cladding is water. Here, the first band becomes fully guided, while the second remains in the leaky region but still aligns with 1550 nm, indicating reduced radiation losses. The upper cladding refractive index significantly influences the effective refractive index and confinement of the propagating modes through the PhCN. Thus, the low or high-frequency band edges of the PhCN can be used to generate the slowly varying background required for modulating t_B and generating the Fano effect at the wavelengths of interest.

D. FDTD simulations from the PhCN varying cladding refractive index

The analysis presented in the manuscript includes numerical simulations of the influence of N_H on the asymmetry for a fixed value of g_y = 0.25 µm. Complementary simulations were also conducted to investigate the influence of g_y on the shape of the resonance for a fixed value of N_H = 5 and L_c. The values of $g_y$were mapped from 0.15 µm to 0.45 µm in steps of 0.1 µm, as shown in Fig. S7. Thus, the correspondence between g_y for tunning Q_c, and N_H for modulating t_B was verified.

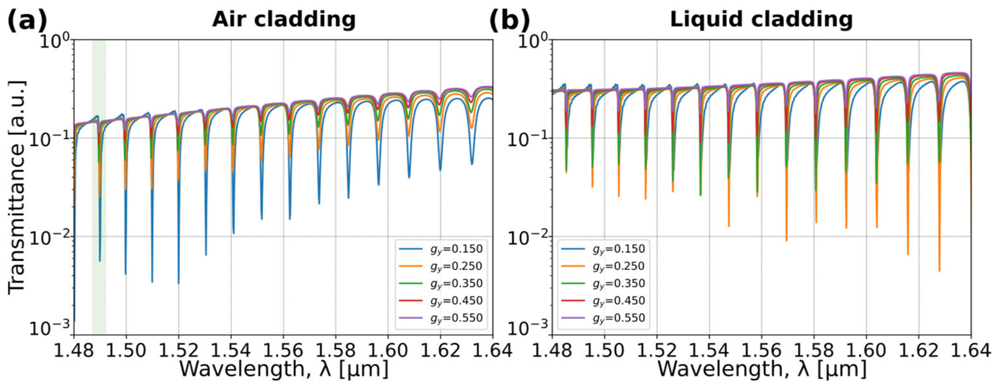

Fig. S7. Rectangular-slot PhCN-MRR designed with fixed R_{MRR}, $N_H = 5$, and $L_C = N_H a$. (a-b) 3D-FDTD calculated transmittance spectra of the proposed device for varying gap size g_y with fixed L_c and N_H when the upper cladding is (a) air and (b) DIW. Zoom in version to the wavelength range of the available source.

All FDTD simulations were conducted using MEEP [27] to support the design and interpretation of the PhCN-MRR device behavior under both air- and DIW-clad conditions. To ensure numerical accuracy and reproducibility, we adopted a uniform mesh grid. A fine mesh size of 30px/μm was applied in all regions, such as the Si_3N_4 waveguides, PhCN, and coupling gap. Besides, subpixel smoothing features were enabled for improved accuracy.

Perfectly Matched Layer (PML) boundary conditions were used in all directions, with 1μm thickness, optimized for convergence in previous simulations for the isolated resonator. A Courant factor of 0.5 was selected to maintain temporal stability. The total simulation time window was set depending on the cladding scenario, based on the full decay of resonant fields (<10-9). Moreover, a padding distance of at least 2μm was set in all directions. Symmetry was applied, only when the resonator was isolated.

Convergence was verified through mesh refinement and extended simulation time for the isolated resonator. Transmittance and reflectance spectrum were calculated by doing two runs, one with and one without the scatterers. Thus, the transmitted flux is normalized by the incident power in the coupled waveguide. The flux monitors were set to get at least 500 points in the target spectral range (1.35 to 1.75μm), depending on if resonant features were present or not, such as the isolated PhCN.

E. Fabrication of the Si_3N_4 PhCN-MRR

Fig. S8 presents the reference SEM pictures from which Fig. 4 and Fig. 1(a), in the main manuscript is generated. Fig. S8(a) the top view of the fabricated device MRR, while Fig. S8(b) details the PhCN in the coupling region.

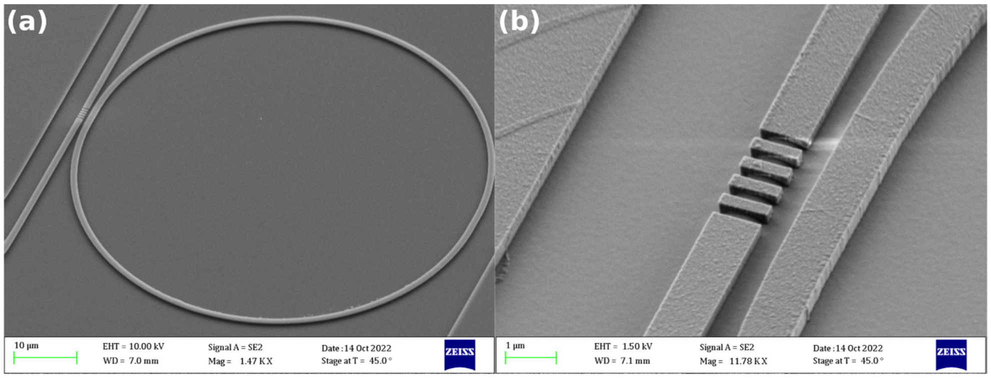

Fig. S8. Source images of Scanning Electron Microscope images of the fabricated device on a 0.3 µm thick Si_3N_4 slab. (a) Lateral view of the fabricated rectangular air-based slot PhCN-MRR. (b) Detail of the coupling region

F. PhCN-MRR characterization

Fig. S9 illustrates the experimental setup developed for the characterization of the fabricated devices. Two configurations were implemented: the first utilized a broadband laser source connected to an optical spectrum analyzer (OSA), while the second employed a semiconductor tunable laser paired with a photodetector. The light source polarization was adjusted to excite the desired waveguide mode, specifically the TE-like mode in this case. A set of lenses was then used to collimate and focus the light onto the input waveguide of the device under test (DUT). The output light was collected using an identical set of lenses, which directed the beam to an optical fiber connected to the detection system. The detection was performed using either the OSA or the photodetector. All measurements were carried out on a thermally stabilized holder maintained at 20°C (±0.02) to minimize the effect of temperature fluctuations in the environment.

Fig. S10 provides a closer view of the setup described in Fig. S9, specifically for the experiments conducted with a DIW-cladding. For device characterization, 10 µL drops of deionized water were applied. Using the imaging system integrated into the setup, each iteration ensured that the drop fully covered the device. After each measurement, the device was rinsed with IPA to ensure proper cleaning and maintain reproducibility.

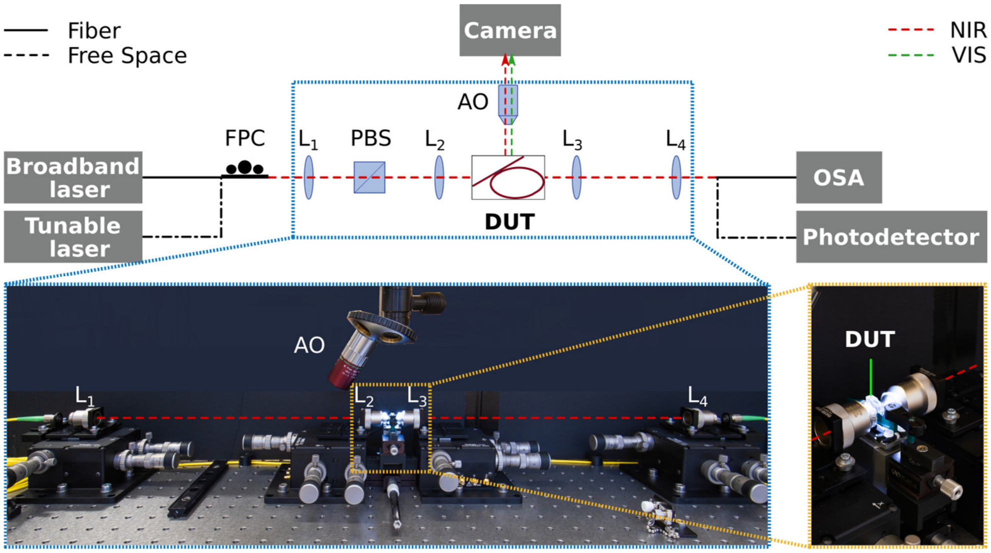

Fig. S9. Setup for the characterization of the proposed Si_3N_4 devices. L_1 and L_4 are collimation lenses with 10X magnification, while L_2 and L_3 are focusing lenses with 60X magnification. AO refers to the apochromatic microscope objective which is assembled with tube lenses and connected to a SWIR camera. FPC is the fiber polarization controller, and PBS is the polarization beam splitter. DUT denotes the device under test. The green dashed line represents the free space light path for visible wavelengths, while the red dashed lines indicate the path for NIR wavelengths. Filled and point dashed lines indicate the two-alternative path for fibers tested.

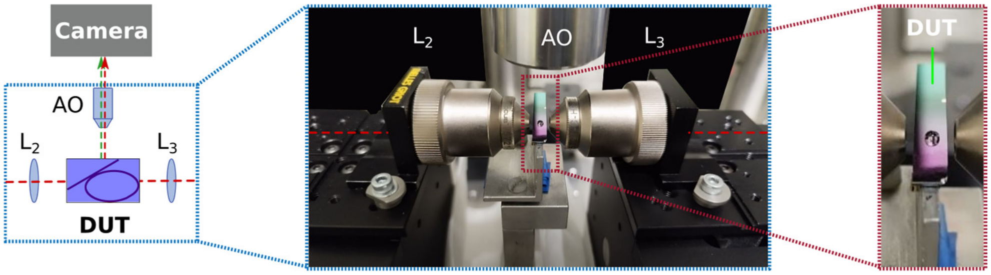

Fig. S10. Schematic and experimental setup for characterizing the proposed device under test (DUT) in an aqueous environment using drop-cast measurements. L_2 and L_3 are 60X magnification focusing lenses. An apochromatic microscope objective (AO), coupled with tube lenses, is connected to a SWIR camera to monitor the droplet coverage on the DUT. A detailed zoomed view of the droplet covering the fabricated device is also shown.

References

1. W. Zhang, W. Li, and J. Yao, "Optically tunable Fano resonance in a grating-based Fabry–Perot cavity-coupled microring resonator on a silicon chip," Opt. Lett. **41**, 2474–2477 (2016).
2. Z. Zhang, G. I. Ng, T. Hu, H. Qiu, X. Guo, W. Wang, M. S. Rouifed, C. Liu, and H. Wang, "Conversion between EIT and Fano spectra in a microring-Bragg grating coupled-resonator system," Appl. Phys. Lett. **111**, 081105 (2017).
3. M. D. Carlo, F. D. Leonardis, F. Dell'Olio, Y. Ding, and V. M. N. Passaro, "Dissipative coupling in a Bragg-grating-coupled single resonator with Fano resonance for anti-PT-symmetric gyroscopes," Opt. Express **32**, 5932–5942 (2024).
4. F. Peng, Z. Wang, G. Yuan, L. Guan, and Z. Peng, "High-Sensitivity Refractive Index Sensing Based on Fano Resonances in a Photonic Crystal Cavity-Coupled Microring Resonator," IEEE Photonics J. **10**, 1–8 (2018).

5. L. Gu, H. Fang, J. Li, L. Fang, S. J. Chua, J. Zhao, and X. Gan, "A compact structure for realizing Lorentzian, Fano, and electromagnetically induced transparency resonance lineshapes in a microring resonator," Nanophotonics **8**, 841–848 (2019).
6. L. Gu, L. Fang, H. Fang, J. Li, J. Zheng, J. Zhao, Q. Zhao, and X. Gan, "Fano resonance lineshapes in a waveguide-microring structure enabled by an air-hole," APL Photonics **5**, 016108 (2020).
7. C. Zhang, G. Kang, Y. Xiong, T. Xu, L. Gu, X. Gan, Y. Pan, and J. Qu, "Photonic thermometer with a sub-millikelvin resolution and broad temperature range by waveguide-microring Fano resonance," Opt. Express **28**, 12599–12608 (2020).
8. L. Fang, L. Gu, J. Zheng, Q. Zhao, X. Gan, and J. Zhao, "Controlling Resonance Lineshapes of a Side-Coupled Waveguide-Microring Resonator," J. Light. Technol. **38**, 4429–4434 (2020).
9. J. H. Mendoza-Castro, A. S. Vorobev, S. Iadanza, B. Lendl, L. O'Faolain, and M. Grande, "Enhanced Fano resonances in a silicon nitride photonic crystal nanobeam-assisted micro ring resonator for dual telecom band operation," Opt. Express **32**, 13197–13207 (2024).
10. C.-Y. Chao and L. J. Guo, "Biochemical sensors based on polymer microrings with sharp asymmetrical resonance," Appl. Phys. Lett. **83**, 1527–1529 (2003).
11. H. Yi, D. S. Citrin, and Z. Zhou, "Highly sensitive silicon microring sensor with sharp asymmetrical resonance," Opt. Express **18**, 2967–2972 (2010).
12. Q. Hong, J. Jiang, S. Zhou, G. Xia, P. Xu, M. Zhu, W. Xu, J. Zhang, and Z. Zhu, "Silicon-Based On-Chip Tunable High-Q-Factor and Low-Power Fano Resonators with Graphene Nanoheaters," Nanomaterials **13**, 1636 (2023).
13. H. Li, L. Lu, G. Chen, S. Wang, J. Ou, and L. Zhu, "Fano Resonance Thermo-Optic Modulator Based on Double T-Bus Waveguides-Coupled Micro-Ring Resonator," Photonics **11**, 255 (2024).
14. Z. Tu, D. Gao, M. Zhang, and D. Zhang, "High-sensitivity complex refractive index sensing based on Fano resonance in the subwavelength grating waveguide micro-ring resonator," Opt. Express **25**, 20911–20922 (2017).
15. K. Y. Yang, J. Skarda, M. Cotrufo, A. Dutt, G. H. Ahn, M. Sawaby, D. Vercruysse, A. Arbabian, S. Fan, A. Alù, and J. Vučković, "Inverse-designed non-reciprocal pulse router for chip-based LiDAR," Nat. Photonics **14**, 369–374 (2020).
16. A. C. Ruege and R. M. Reano, "Multimode waveguide-cavity sensor based on fringe visibility detection," Opt. Express **17**, 4295–4305 (2009).
17. D. Ding, M. J. A. de Dood, J. F. Bauters, M. J. R. Heck, J. E. Bowers, and D. Bouwmeester, "Fano resonances in a multimode waveguide coupled to a high-Q silicon nitride ring resonator," Opt. Express **22**, 6778–6790 (2014).
18. T. Yuan, X. Wang, J. Wu, H. Li, Y. Chen, and X. Chen, "Frequency-Space Selective Fano Resonance Based on a Micro-Ring Resonator on Lithium Niobate on Insulator," Laser Photonics Rev. **n/a**, 2400457 (n.d.).
19. M. Heuck, P. T. Kristensen, Y. Elesin, and J. Mørk, "Improved switching using Fano resonances in photonic crystal structures," Opt. Lett. **38**, 2466–2468 (2013).
20. S. Fan, W. Suh, and J. D. Joannopoulos, "Temporal coupled-mode theory for the Fano resonance in optical resonators," JOSA A **20**, 569–572 (2003).
21. D. Bekele, Y. Yu, K. Yvind, and J. Mork, "In-Plane Photonic Crystal Devices using Fano Resonances," Laser Photonics Rev. **13**, 1900054 (2019).
22. W. Bogaerts, P. De Heyn, T. Van Vaerenbergh, K. De Vos, S. Kumar Selvaraja, T. Claes, P. Dumon, P. Bienstman, D. Van Thourhout, and R. Baets, "Silicon microring resonators," Laser Photonics Rev. **6**, 47–73 (2012).
23. C. A. Barrios, B. Sánchez, K. B. Gylfason, A. Griol, H. Sohlström, M. Holgado, and R. Casquel, "Demonstration of slot-waveguide structures on silicon nitride / silicon oxide platform," Opt. Express **15**, 6846–6856 (2007).
24. P. Cheben, R. Halir, J. H. Schmid, H. A. Atwater, and D. R. Smith, "Subwavelength integrated photonics," Nature **560**, 565–572 (2018).
25. S. G. Johnson and J. D. Joannopoulos, "Block-iterative frequency-domain methods for Maxwell's equations in a planewave basis," Opt. Express **8**, 173–190 (2001).
26. J. D. Joannopoulos, ed., *Photonic Crystals: Molding the Flow of Light*, 2nd ed (Princeton University Press, 2008).
27. A. F. Oskooi, D. Roundy, M. Ibanescu, P. Bermel, J. D. Joannopoulos, and S. G. Johnson, "Meep: A flexible free-software package for electromagnetic simulations by the FDTD method," Comput. Phys. Commun. **181**, 687–702 (2010).
